# Evaluating mobile-based data collection for crowdsourcing behavioral research

**DOI:** 10.3758/s13428-025-02618-1

**Published:** 2025-02-28

**Authors:** Dennis T. Esch, Nikolaos Mylonopoulos, Vasilis Theoharakis

**Affiliations:** 1 Cranfield School of Management, Cranfield University, College Road, Cranfield, Bedfordshire, MK43 0AL UK; 2 Alba Graduate Business School, The American College of Greece, Athens, Greece

**Keywords:** Data quality, Online research, Mobile, Crowdsourcing, Attention checks, Pollfish, MTurk, Prolific, Qualtrics

## Abstract

Online crowdsourcing platforms such as MTurk and Prolific have revolutionized how researchers recruit human participants. However, since these platforms primarily recruit computer-based respondents, they risk not reaching respondents who may have exclusive access or spend more time on mobile devices that are more widely available. Additionally, there have been concerns that respondents who heavily utilize such platforms with the incentive to earn an income provide lower-quality responses. Therefore, we conducted two studies by collecting data from the popular MTurk and Prolific platforms, Pollfish, a self-proclaimed mobile-first crowdsourcing platform, and the Qualtrics audience panel. By distributing the same study across these platforms, we examine data quality and factors that may affect it. In contrast to MTurk and Prolific, most Pollfish and Qualtrics respondents were mobile-based. Using an attentiveness composite score we constructed, we find mobile-based responses comparable with computer-based responses, demonstrating that mobile devices are suitable for crowdsourcing behavioral research. However, platforms differ significantly in attentiveness, which is also affected by factors such as the respondents’ incentive for completing the survey, their activity before engaging, environmental distractions, and having recently completed a similar study. Further, we find that a stronger system 1 thinking is associated with lower levels of attentiveness and acts as a mediator between some of the factors explored, including the device used and attentiveness. In addition, we raise a concern that most MTurk users can pass frequently used attention checks but fail less utilized measures, such as the infrequency scale.

**Supplementary information:**

The online version contains supplementary material available at 10.3758/s13428-025-02618-1.

## Introduction

Online behavioral research has thrived over the last two decades, thanks in no small part to the proliferation of crowdsourcing platforms such as Amazon Mechanical Turk (MTurk) and Prolific (Berinsky et al., 2012; Buhrmester et al., 2011; Litman et al., 2017; Palan & Schitter, 2018; Peer et al., 2017). These widely utilized platforms have dramatically reduced the resources required for data collection, thus revolutionizing how researchers across disciplines collect data (Chandler & Shapiro, 2016; Zhou & Fishbach, 2016). However, these platforms are not without criticism, with various concerns being raised, such as the non-naivety of respondents (e.g., Chandler et al., 2014; Chandler & Paolacci, 2017; Ford, 2017; Sharpe Wessling et al., 2017). Particularly for MTurk, there are stern warnings about the quality of data produced by participants whose main incentive is to use the platform as a source of income (Peer et al., 2021), with evidence that standard attention checks fail to sufficiently filter poor responses (Thomas & Clifford, 2017; Webb & Tangney, 2022). This paper seeks to contribute to this literature by further exploring such concerns.

One of the appeals of crowdsourcing platforms was to move away from the reliance on student samples (Ferber, 1977; Henry, 2008; Knoll, 2016; Norenzayan & Heine, 2005; Peterson, 2001; Petty & Cacioppo, 1996; Sears, 1986) and to broaden the respondent pool (Chandler et al., 2013). While this has been generally achieved (Goodman et al., 2013; Goodman & Paolacci, 2017; Paolacci & Chandler, 2014), widely used crowdsourcing platforms primarily reach computer-based participants. Nonetheless, only 47.1% of households worldwide have a computer (Statista, 2021b), in contrast to 73.5% having a smartphone (Statista, 2021a). Therefore, given the overall prevalence of smartphones, mobile devices^1^ have the potential to offer a broader reach to a larger population. As Konitzer et al. (2016) argue, data collection via mobile devices represents a new mode on top of the established face-to-face, mail, telephone, and web modes.

However, the context of use and attitudes between personal computers and smartphone users may differ, influencing response quality. On the one hand, problematic smartphone use (Mylonopoulos & Theoharakis, 2021; Soror et al., 2015) has been positively associated with addiction-like symptoms and ADHD traits (Panagiotidi & Overton, 2022), raising the question whether smartphone-based respondents demonstrate the necessary attention when completing surveys. Further, the cognitive response system utilized may also influence response quality, with concerns raised that mobile users more strongly utilize system 1 thinking (King et al., 2015), which tends to allocate fewer resources to their task (Kahneman, 2011), potentially resulting in lower attentiveness.

On the other hand, mobile users may also reach a state of flow when they are fully immersed in their device and feel an energized focus that is positive and “aligned with the task at hand” (Khang et al., 2013). Such a focus would imply that mobile respondents would be quite attentive when presented with a survey, thus providing high-quality data. One could also argue that such a flow could be negative if the respondent were interrupted from using their favorite app to complete a survey. These concerns align with Antoun et al. (2017), who nonetheless find that smartphone respondents can provide high-quality responses. Whether one or the other phenomenon prevails is an empirical question that may depend on factors such as respondent circumstances, incentives, the surrounding environment, and the activity they performed before completing the survey, which can vary considerably and affect data quality^2^. For example, when examining response quality, one would also need to account for the respondent’s ability to concentrate on the task at hand, as distractions in the environment and time pressure may not allow them to remain focused.

For these reasons, we extend previous platform comparisons (Peer et al., 2017, 2021) by adding in our examination Pollfish, a self-proclaimed mobile-first crowdsourcing platform that has received responses from more than 250 million individuals in more than 160 countries (Pollfish, 2022a). According to the company, Pollfish provides AI-based algorithmic data quality checks to return only “valid” responses, while questionnaires are integrated natively into partner apps (Pollfish, 2022b). In contrast to MTurk and Prolific, Pollfish requires researchers to use its own survey development tool, limits the number of questions allowed^3^, reviews and approves the questionnaire as part of its standard process, and provides respondent demographics. Qualtrics, an additional platform we examine, has also developed a market research panel, which aims to be “always representative” (Qualtrics, 2024). While the company does not describe how it recruits its audience, executing a survey requires engagement with an account team that manages the process, i.e., it is not as automated and quick as the process with MTurk, Prolific, or Pollfish. According to Qualtrics^4^, they also pause data collection to “scrub” responses before delivering them to researchers; they remove gibberish responses, straight-liners, duplicate respondents, laggards, and speeders while carrying out security checks for bots. Given that both Pollfish and Qualtrics recruit mobile-based respondents and aim to secure data quality differently, we examine these platforms along with the extensively utilized and studied MTurk and Prolific.

This paper poses the following research questions: (i) does response quality from predominantly mobile platforms differ from that of popular, primarily computer-based platforms? (ii) does response quality within the same platform differ between mobile-based users when compared to responses from computer-based users? (iii) does response quality differ depending on user context and circumstances, such as incentive received, activity engaged before responding to the survey, distractions, ability to concentrate, and time pressure?

## Study 1

### Method

#### Sampling and participants

To answer our research questions, we ran the same online study on five different platforms: MTurk^5^, Prolific, Qualtrics audience panel, and twice on Pollfish – once through its native survey development interface and once through its third-party channel, which we consider as a different platform^6^. We collected data on Pollfish via these two distinct routes since its native survey interface includes AI data quality filters before delivering responses to researchers. This feature was unavailable through the third-party Pollfish channel, allowing us to evaluate the platform’s data quality before and after their AI filtering intervention. We call data collected through the native Pollfish interface *Pollfish* and data collected via the third-party Pollfish channel *Pollfish 3*^*rd*^* party* (*Pollfish 3P*). At the time, the Qualtrics audience panel data made all responses available on the system but indicated the responses they deemed poor. We call the approved data *Qualtrics* and the total set of responses *Qualtrics Raw*. Qualtrics was used as the survey development tool across all platforms except *Pollfish,* which requires its own survey tool.

Our target sample size was 400 participants per platform, leading to an overall target sample size of 2000 participants. The sample size was determined before any data analysis, based on the review of a previous platform comparison (Peer et al., 2017), which had a minimum sample size of 195. We doubled the sample size they achieved per platform to increase statistical power. Following previous similar studies, our study was available only to US residents. It was launched on a Sunday morning in late January 2021, except for the Qualtrics panel study, which was launched on a Sunday morning in late April 2021. Overall, 2096 respondents completed the study (MTurk: *n* = 407; Prolific: *n* = 401; Pollfish: *n* = 400; Pollfish 3P: *n* = 421; Qualtrics: *n* = 441; Qualtrics Raw: *n* = 467). The cost per response was as follows: $3 for Pollfish, $0.95 for Pollfish 3P, $1.45 for MTurk, $1.21 for Prolific, and $3.88 for Qualtrics. While platforms may have offered the possibility to target specific groups or even deliver samples representative of the population at an additional cost, we did not select any such options because we wanted to be consistent across platforms, as one can apply weights on data post hoc to make them representative of the population. That means no pre-filters, other than the US residency requirement, were used on any platform.

##### Response rates and time to completion

As shown in Table 1, the number of responses collected was similar across all platforms. Still, dropout rates varied dramatically, with 3% for MTurk and Prolific, almost 20% for the sample recruited via Pollfish 3P, and more than 50% for the Qualtrics panels and Pollfish (based on the completion rate reported by the platform). The considerable dropout rates for Pollfish and Qualtrics raise concerns as attrition can significantly affect empirical studies (Zhou & Fishbach, 2016). All following analyses are conducted only on those participants who completed the study.

**Table 1:** Sample sizes, dropout rates, completion time, and device used

Platform	Starts	Completions	Dropout	Completion Time (mins)	Mobile		Computer
MTurk	420	407	3.1%	84		3.9%	96.1%
Prolific	413	401	2.9%	130		20.7%	79.3%
Pollfish	968	400	58.7%	930		71.5%	28.5%
Pollfish 3P	509	421	17.3%	901		71.3%	28.7%
Qualtrics	929	441	52.5%	1327		66.0%	34.0%
Qualtrics Raw^a^	955	467	51.1%	329		66.8%	33.2%

While for MTurk, Prolific, Pollfish 3P and Qualtrics panels, the dropout rate is derived from the number of respondents who started and did not complete the study, for Pollfish, starts are inferred from the completion rate provided by the platform

^a^We report results for Qualtrics rather than Qualtrics Raw throughout the paper as this is what the platform ultimately delivers. We report Qualtrics Raw results on top of this, where there are significant deviations from the Qualtrics results

We reached the target sample size for all platforms before our cutoff point of 1 week (see Table 1). While the sample size was reached very quickly on MTurk and Prolific, the Qualtrics sample took the longest time, with Qualtrics Raw, Pollfish 3P, and Pollfish in between. We attribute the extensive time taken by Qualtrics to data collection pauses for data scrubbing.

A comparison of participation time showed significant differences (*F* (4, 2065) = 3.933, *p* = .004, *f* = .087), with pairwise comparisons (Tukey HSD) indicating that Pollfish was significantly different from MTurk (*p* < .05). A follow-up analysis revealed that participation time across the platforms examined did not differ based on the respondent’s device to take part (*F* (3, 1655) = 1.165, *p* = .322, *f* = .046).

##### Device usage

We captured the device used based on functionality available in the Qualtrics survey software and Pollfish’s native survey platform. We find that there are significant differences in terms of device used across platforms (c^2^ (4) = 770.403, *p* < .001, φ = .610), with MTurk and Prolific respondents taking part predominantly using a personal computer, while Pollfish and Qualtrics respondents predominantly participated using mobile devices (see Table 1).

Given the distinct difference between MTurk/Prolific and Pollfish/Qualtrics regarding the device used to access the study, we examine data quality indicators by investigating whether potential differences are driven by the device type used. However, given that MTurk’s interface is designed for computer-based users, we received an exceedingly small number of mobile users, which did not allow us to follow this approach for the MTurk sample.

#### Procedure

Participants were invited to a study billed to examine the behavior of online survey participants. The questionnaire comprised of three blocks of questions and tests, broadly following the research strategy of similar studies (Goodman et al., 2013; Paolacci & Chandler, 2014). The first block consisted of a battery of questions measuring widely used constructs in psychology focusing on individual differences (Big Five personality traits, self-esteem, self-control, need for cognition, social desirability). The second block consisted of prominent psychology tests (Asian disease problem, Linda problem, Cognitive Reflection Test). The last block covered participants’ usage of online study platforms and common demographic questions. While the order of the blocks was fixed, the items within the constructs and tests of the first two blocks were fully randomized. Throughout the different blocks, three different attention check questions were integrated.

#### Materials^7^

All data and materials are available as supplementary materials, and the study was not preregistered. To investigate individual participant differences across different platforms, we included several well-established and widely used measures such as the ten-item personality inventory (TIPI; Gosling et al., 2003), a very brief measure of the Big Five personality inventory (John et al., 2008), the Rosenberg Self-Esteem Scale (RSES; Rosenberg, 1965), as well as very brief versions of the Self-Control (Maloney et al., 2012) and Need for Cognition Scales (NFC; de Holanda Coelho et al., 2020). Aside from their widespread use, these measures were selected because researchers had previously adopted them when assessing online behavioral research platforms (Goodman et al., 2013; Peer et al., 2014, 2017). All constructs were measured using a seven-point Likert scale from strongly disagree (1) to strongly agree (7). To assess respondents’ likelihood to engage in socially desirable behavior, we used the short form of the Marlowe–Crowne Social Desirability Scale developed by Strahan and Gerbasi (1972), as this has been shown to be superior to the original scale and other short forms (Fischer & Fick, 1993).

Following Paolacci et al. (2010) and Goodman et al. (2013), we also included three widely used judgment and decision-making problems to assess how far they replicate across our different platforms. Firstly, we used the so-called Asian disease problem (Tversky & Kahneman, 1981) as an example of the impact of framing on choice. Participants were confronted with a scenario in which a country faces an unusual disease, and different courses of action are available, which are framed in terms of the number of people either dying or surviving. Secondly, we used the Linda problem (Tversky & Kahneman, 1983) as an example of the conjunction fallacy, i.e., people’s tendency to regard the combination of events as more probable than any of the events on their own. Thirdly, we included the cognitive reflection test (Frederick, 2005) to assess the strength of people’s system 1 (Stanovich, 1999), i.e., how far they are driven by their more automatic, emotional reaction to stimuli. Beyond this, we captured how frequently respondents participated in research studies in return for rewards using the frequency categories used by Peer et al. (2017). Lastly, we collected several demographic variables (age, gender, race, education, employment status, household income)^8^. We also included three attention check questions, two of which were bogus items (Meade & Craig, 2012), blending them with the surrounding items of the previously described scales. The last attention check was an instructional manipulation check (Oppenheimer et al., 2009) placed at the end of the study.

### Results

#### Differences across platforms

##### Attention

**Fig. 1:**
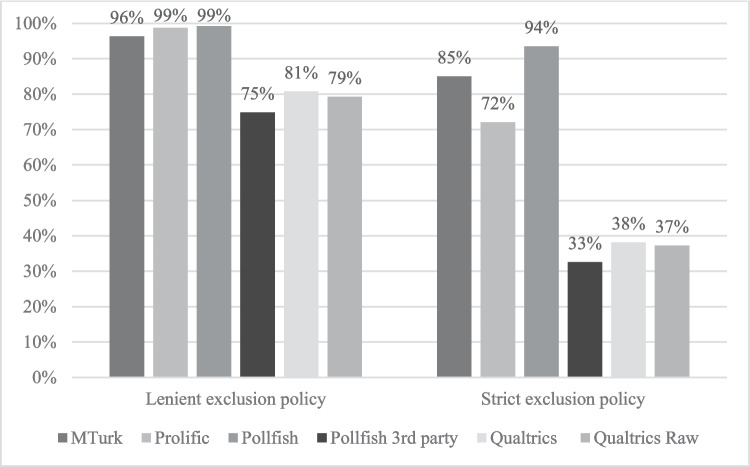
Percentage of participants retained based on different exclusion policies

We gauged participant attention by integrating three attention check questions throughout the study and followed Peer et al. (2017) by considering two exclusion policies, using the criteria of Meade and Craig (2012). In the lenient exclusion policy, participants were retained if they had passed any two out of the three attention checks. In the strict exclusion policy, participants were retained only if they had passed all attention checks. Figure 1 shows the impact of the different exclusion policies on the sample percentage retained across the platforms examined. Two overall groups emerge with the lenient exclusion policy: for MTurk, Prolific, and Pollfish, barely any participants are dropped, while for Pollfish 3P and Qualtrics, between a fifth and a quarter of participants are dropped. These differences become more pronounced under the strict exclusion policy. Although a critical decision for researchers is how to handle responses from inattentive participants (Berinsky et al., 2016), for the remainder of the paper, results are based on the total sample without any exclusions except where otherwise stated.

To confirm the significance of these differences, we also examine whether the average number of attention checks passed by participants differed across platforms (Table 18 in the appendix). There were significant differences (*F*(4, 2065) = 169.81, *p* < .001, *f* = .574), and pairwise comparisons (Tukey HSD) showed that Pollfish respondents passed the most attention checks, similar to MTurk respondents, which were in turn similar to Prolific respondents. Qualtrics respondents passed significantly fewer attention checks, and Pollfish 3P respondents had the worst pass rates (all reported differences *p* < .05)^9^.

We also examined differences in pass rates across the three different attention checks used. The two bogus items were randomized in the first block, while the third was an instructional manipulation check with a fixed position at the end of the study. In general, pass rates were much higher across the board for the two bogus items than the instructional manipulation check, which was best for differentiating between platforms (see Table 19 in the appendix for pass rates per attention check across platforms).

##### Individual differences

Respondents differed significantly across platforms in terms of extraversion (*F*(4, 2065) = 13.371, *p* < .001, *f* = .161), agreeableness (*F*(4, 2065) = 6.748, *p* < .001, *f* = .114), conscientiousness (*F*(4, 2065) = 15.444, *p* < .001, *f* = .173), neuroticism (*F*(4, 2065) = 14.090, *p* < .001, *f* = .165), openness to experiences (*F*(4, 2065) = 5.915, *p* < .001, *f* = .107), self-esteem (*F*(4, 2065) = 15.749, *p* < .001, *f* = .175), self-control (*F*(4, 2065) = 16.416, *p* < .001, *f* = .178), and need for cognition (*F*(4, 2065) = 3.643, *p* = .001, *f* = .084). MTurk and Prolific participants were lowest in terms of extraversion, MTurk, and Pollfish participants were particularly high in agreeableness, conscientiousness, and self-esteem, while MTurk participants scored – on average – much higher on neuroticism than other samples (Table 20 in the appendix). Notably, the AI filtering employed by Pollfish appears to retain more conscientious respondents, given that the unfiltered Pollfish 3^rd^ party sample is among the least conscientious overall.

##### Reliability

We then compared Cronbach’s alphas to measure internal reliability for the utilized Self-Esteem (SE), Self-Control, and Need for Cognition Scales across platforms. Overall, scale reliability did vary between platforms (see Table 2), which we tested using the Hakstian and Whalen (1976) procedure but also re-confirmed using Monte Carlo simulations (Good, 2000). While reliabilities are acceptable in most parts (see Table 2 and Table 21 in the appendix), the Qualtrics, Qualtrics Raw, and Pollfish 3P samples for NFC do not pass the 0.7 threshold. Overall, reliabilities are generally higher for the MTurk sample, followed by Prolific and then Pollfish. The lowest reliabilities are typically found for the Pollfish 3P and Qualtrics samples that appear similar in behavior as they do not significantly differ for two (SE, NFC) of the three scale reliabilities.

**Table 2:** Cronbach’s alpha values per platform and measure and chi-squared values of reliability differences between platforms

Platform	Overall α	MTurk	Prolific	Pollfish	Pollfish 3P
	Self-Esteem				
MTurk	0.935				
Prolific	0.932	0.251 (.617)			
Pollfish	0.909	10.231 (.001)	7.252 (.007)		
Pollfish 3P	0.863	51.298 (< .001)	44.247 (< .001)	15.713 (< .001)	
Qualtrics	0.864	50.975 (< .001)	43.822 (< .001)	15.115 (< .001)	0.017 (.897)
Qualtrics Raw	0.858	59.622 (< .001)	51.714 (< .001)	19.488 (< .001)	0.117 (.732)
	Need for Cognition				
MTurk	0.925				
Prolific	0.870	24.427 (< .001)			
Pollfish	0.811	68.302 (< .001)	11.806 (.001)		
Pollfish 3P	0.661	177.401 (< .001)	76.061 (< .001)	28.606 (< .001)	
Qualtrics	0.661	183.452 (< .001)	78.356 (< .001)	29.387 (< .001)	0.000 (-)
Qualtrics Raw	0.658	193.285 (< .001)	82.775 (< .001)	31.335 (< .001)	0.008 (.928)
	Self-Control				
MTurk	0.879				
Prolific	0.835	8.480 (.004)			
Pollfish	0.808	18.680 (< .001)	2.023 (.155)		
Pollfish 3P	0.787	28.672 (< .001)	5.857 (.016)	0.959 (.327)	
Qualtrics	0.720	63.274 (< .001)	25.185 (< .001)	12.737 (< .001)	6.824 (.009)
Qualtrics Raw	0.714	68.876 (< .001)	28.168 (< .001)	14.690 (< .001)	8.202 (.004)

*Note:* Chi-squared values and *p* values in parentheses

We also examine how our attention checks may influence the reliability of constructs by comparing reliabilities between the portion of the sample that passes our strict exclusion test vs. the remaining sample. From the 18 cases examined (Table 22 in the appendix), 14 reliabilities improve under strict exclusion conditions.

##### Reproducibility

We examined the reproducibility of known effects across platforms. The basic pattern of all effects (Asian disease problem as an example of the framing effect, Linda problem as an example of the conjunction fallacy, and the cognitive reflection test) was replicated, but the strength of the effects varied. Results for the framing problem participants faced (Asian disease problem) differed significantly across platforms. Table 3 reports the effect sizes, with a higher Cramer’s V indicating that participants are more susceptible to how the options were framed. The framing effect was, therefore, most pronounced for Prolific participants and least pronounced for Pollfish 3^rd^ party and Qualtrics respondents. Further testing of Cramer’s V pairwise differences using Monte Carlo permutations indicates that Prolific respondents tend to be more susceptible to the framing effect (see Table 3).

**Table 3:** Cramer’s V for the Asian disease problem across platforms and exclusion policies

	Platform	*Cramer's V*	Prolific	Pollfish 3P	Pollfish	Qualtrics
No exclusion	MTurk	*0.355*	.014	.046	.556	.082
	Prolific	*0.505*		< .001	.060	< .001
	Pollfish 3P	*0.211*			.006	.656
	Pollfish	*0.390*				.022
	Qualtrics	*0.236*				
Lenient exclusion	MTurk	*0.368*	.018	.040	.734	.204
	Prolific	*0.504*		< .001	.044	< .001
	Pollfish 3P	*0.225*			.034	.488
	Pollfish	*0.386*				.098
	Qualtrics	*0.276*				
Strict exclusion	MTurk	*0.347*	.020	.488	.686	.880
	Prolific	*0.524*		.020	.048	.030
	Pollfish 3P	*0.282*			.304	.610
	Pollfish	*0.374*				.630
	Qualtrics	*0.334*				

*Note:* Two-tailed *p* values of pairwise differences after 1000 Monte Carlo permutations

In line with previous studies, respondents across all platforms fell prey to the conjunction fallacy: more people regarded the less likely option (“Linda is a bank teller and is active in the feminist movement.”) to be more probable than the more likely option (“Linda is a bank teller.”). While there are significant differences in terms of the Linda problem (c^2^ (4) = 12.124, *p* < .001, φ = .077), the overall pattern was similar (ranging from 83% of Pollfish respondents to 74% of Qualtrics respondents regarding the less likely option to be more probable).

For the cognitive reflection test, the differences are most dramatic (*F*(4, 2065) = 184.552, *p* < .001, *f* = .277). Participants recruited via Pollfish and Qualtrics demonstrated a highly active system 1, while MTurk participants exhibited the strongest system 2 response, followed by Prolific participants, who showed a less pronounced but still elevated system 2 response (see Table 23 in the appendix).

##### Social desirability

We included a measure of social desirability as an additional data quality indicator, where fewer socially desirable responses indicate higher data quality (Peer et al., 2014). There were significant differences in terms of how participants responded to the social desirability scale across platforms (*F*(4, 2065) = 23.604, *p* < .001, *f* = .214), with pairwise comparisons (Tukey HSD, all reported differences *p* < .05) indicating that MTurk (*M* = 2.74, *SD* = 2.01) and Prolific participants (*M* = 2.64, *SD* = 1.58) engaged in less socially desirable responding than Pollfish (*M* = 3.41, *SD* = 1.2), Pollfish 3^rd^ party (*M* = 3.33, *SD* = 1.65) and Qualtrics participants (*M* = 3.44, *SD* = 1.54). The pattern persisted with the lenient and strict exclusion policies.

#### Differences across devices

##### Attention

For most platforms (Prolific, Pollfish, Pollfish 3^rd^ party), there were no statistically significant differences in the average attention checks passed based on the device used, except for mobile respondents in both Qualtrics panels who passed fewer attention checks than their computer-based respondents (see Table 18 in the appendix for more details). Overall, we found a significant device * platform interaction (*F* (3, 1655) = 3.65, *p* = .012, *f* = .081).

##### Individual differences

Follow-up analyses indicated no significant differences in the individual differences variables across platforms based on the device used to access the study. The only exception was self-esteem (*F* (3, 1655) = 3.908, *p* = .009, *f* = .086), with Qualtrics computer-based users reporting higher self-esteem than Qualtrics mobile users (*M*_CQ_ = 5.29, *SD*_CQ_ = 1.03 vs. *M*_MQ_ = 4.70, *SD*_MQ_ = 1.24).

##### Reliability

We further tested for any reliability differences depending on the type of device across each platform (Table 21 in the appendix). From the 15 cases examined, we only find two significant differences for the NFC scale, where computer-based users performed better for Prolific and Qualtrics. Interestingly, while Qualtrics is one of these cases, Qualtrics Raw is not, i.e., the data eliminated appear to have an effect. Presumably, the removal of cases was sufficient to make Qualtrics computer users statistically more reliable relative to mobile users. Nonetheless, the great majority of differences between mobile and computer users are not significant, suggesting that mobile devices provide sufficiently reliable responses.

##### Reproducibility

A follow-up analysis indicated no significant device * platform interaction for the Linda problem (c^2^ (4) = 8.504, *p* = .075, φ = .072), i.e., mobile and computer-based respondents similarly fell prey to the conjunction fallacy across platforms.

A follow-up analysis on the cognitive reflection test revealed a significant device * platform interaction (*F* (3, 1655) = 5.752, *p* = .001, *f* = .102), with Prolific, Qualtrics panel and Pollfish 3P mobile respondents showing a stronger system 1 than their computer-based counterparts (pairwise comparisons, all reported differences *p* < .05). This means that respondents of these two platforms were less analytical when using mobile devices than their counterparts using computers (see Table 23 in the appendix).

##### Social desirability

A follow-up analysis indicated that social desirability across platforms did not differ based on the respondent’s device used to participate (*F*(3, 1655) = 0.610, *p* = .609, *f* = .033).

### Summary of study 1 findings

By comparing well-established platforms such as MTurk and Prolific, attracting largely computer-based respondents, with Qualtrics and Pollfish, which attract a substantial proportion of mobile-based respondents, our findings provide significant insights about the impact of the platform and device usage on data quality.

First, when comparing platforms, we found significant differences in terms of attention check pass rates between platforms. Pollfish, the mobile-first platform examined, had the most attentive respondents via its native interface, similar to MTurk and outperforming Prolific. In terms of construct reliability, MTurk and Prolific are consistently better than Pollfish, which nonetheless demonstrated high construct reliability (Cronbach’s alphas higher than 0.8). The other three platforms (Pollfish 3P, Qualtrics, and Qualtrics Raw) follow with mixed results in cases such as NFC.

As Pollfish data through its native interface are significantly better in quality across the board when compared to Pollfish 3P data, the advertised AI-driven fraud prevention algorithms indeed appear to improve data quality. However, researchers are not provided access to the responses that are eliminated through this process, which is problematic as the AI algorithm might systematically exclude particular groups of respondents. It is, therefore, difficult for researchers to make more informed decisions on whether their sample is unbiased or not. Further, while MTurk and Prolific had remarkably low dropout rates, the high dropout rates of Pollfish and Qualtrics raise concerns.

Second, in terms of device usage, our analysis found no substantial difference in attention across most platforms when comparing mobile and computer respondents, except for Qualtrics. Similarly, in 13 out of 15 comparisons, there are no significant differences in terms of construct reliability when comparing mobile and computer-based responses. However, the cognitive reflection test suggests significant differences between mobile and computer-based respondents. Notably, mobile device users on Prolific, Pollfish 3P and Qualtrics were found to more significantly engage in system 1 thinking, suggesting that mobile use might be associated with greater levels of intuitive instead of analytical processing.

These findings provide some answers to our first two research questions. However, concerns remain regarding the efficacy of commonly used attention checks, necessitating the examination of additional relevant measures as suggested by Maniaci and Rogge (2014). Further, more insight is required to clarify not only how device choice influences attentiveness but also the context in which the device is being used. Based on the results of study 1, in study 2 we examine in more detail the measurement of attentiveness, the contextual conditions when participants respond to the study, and the role of system 1 versus system 2 thinking.

## Study 2

### Method

#### Sampling and participants

Similarly to study 1, we ran the same online study on four platforms: MTurk^10^, Prolific, Pollfish, and the Qualtrics audience panel, with a target sample size of 400 US residents per platform. It was launched on a Sunday morning in mid-February 2024. All studies were planned to be available for one week to allow sufficient time to reach participant recruitment targets, but samples were filled much faster on all platforms. Overall, 1,635 respondents completed the study (MTurk: *n* = 429; Prolific: *n* = 400; Pollfish: *n* = 400; Qualtrics: *n* = 406)^11^. The cost per response requested was as follows: $4.4 for Pollfish, $1.86 for MTurk, $2.07 for Prolific^12^, and $4.36 for Qualtrics. We did not utilize the ability offered by platforms to target specific groups or even deliver samples representative of the population at an additional cost.

##### Response rates and time to completion

As shown in Table 4, the number of responses collected was similar across all platforms. Still, dropout rates varied dramatically, ranging from roughly 2% for Prolific to 74% for the sample recruited via Pollfish. All following analyses are conducted only on those participants who completed the study. We reached the target sample size for all samples before our cutoff point of 1 week. Three of the samples were completed within hours, with Pollfish taking less than half the time of Prolific and MTurk – this is a notable difference for Pollfish, which took considerably longer in study 1. In contrast, Qualtrics took just under two days to complete data collection, which we attribute to the extensive time Qualtrics took for data collection pauses due to data scrubbing.

**Table 4:** Sample sizes, dropout rates, completion time, and device used

Platform	Starts	Completions	Dropout	Completion Time (mins)	Mobile	Computer
MTurk	479	429	10.4%	67	5.4%	94.6%
Prolific	407	400	1.7%	124	32.8%	67.3%
Pollfish	1,552	400	74.2%	55	76.3%	23.8%
Qualtrics^a^	471	406	13.2%	2,611	73.2%	26.9%

While for MTurk, Prolific and Qualtrics panels, the dropout rate is derived from the number of respondents who started and did not complete the survey, for Pollfish, starts are inferred from the completion rate provided by the platform

^a^For study 2, either no data points were removed by the platform or these were dropped without indication

##### Device usage

We find significant differences in device usage (c^2^ (3) = 661.163, *p* < .001, φ = .636), and as can be seen in Table 4, MTurk and Prolific respondents predominantly participated using a personal computer, while those recruited via Pollfish and Qualtrics predominantly participated using mobile devices. Given the distinct difference between MTurk/Prolific and Pollfish/Qualtrics regarding the device used to access the study, we examine data quality indicators by investigating whether potential differences are driven by the device type used. However, given its exceedingly small number of mobile users, we again cannot follow this approach for the MTurk sample.

#### Procedure

The procedure for study 2 was identical to study 1. We had three blocks with different content. The order of the blocks was fixed, but the items within the first two blocks were fully randomized. Throughout the different blocks, three different attention check questions were integrated.

#### Materials

All data and materials are available as supplementary materials and study 2 was preregistered (https://aspredicted.org/273v-g5y7.pdf). The materials used in study 2 were identical to study 1 except for the following changes. We excluded measures of self-control and personality and the Asian disease problem. We replaced the original cognitive reflection test by Frederick (2005) with an alternate version by Thomson and Oppenheimer (2016) as many respondents are familiar with the original test and because it has been shown to be confounded with numeracy (Campitelli & Gerrans, 2014; Liberali et al., 2012; Weller et al., 2013). We also use multiple indices of inattentiveness on top of the three attention checks (two bogus items and one instructional manipulation check from study 1), as prior research has shown that they have become less effective due to their widespread use (Thomas & Clifford, 2017), that they can be answered automatically (Pei et al., 2020) and that they do not necessarily identify bots (Webb & Tangney, 2022). More specifically, we utilized a simple self-report measure (Meade & Craig, 2012) and the 11-item infrequency scale (IFS) by Maniaci and Rogge (2014), which states that respondents with higher scores are considered inattentive if they exceed a specific cutoff point. Further, to capture more of the respondent’s circumstances at the time of responding, we include scales on how easy it is for the respondent to concentrate during the survey, their current time constraints, the activity they are engaged in just before answering the survey, their participation incentives, and the distraction level in their environment. Finally, we asked how many similar platforms respondents have signed up to, whether they had signed up to participate in studies, and whether they had taken part in a similar study recently.

### Results

#### Differences across platforms

##### Attention

We gauged participant attention using three different approaches. Firstly, we aggregated the three different attention-check questions throughout the study. Using the criteria of Meade and Craig (2012), we examined whether the average number of attention checks passed by participants differed across platforms. There were significant differences (*F*(3, 1631) = 60.24, *p* < .001, *f* = .333), with MTurk and Qualtrics respondents scoring similarly in terms of attention checks, while Pollfish and Prolific respondents scored progressively better (see Table 5).

**Table 5:** Attention checks passed and IFS score (pairwise comparisons)

	Attention checks		IFS score	
Platform	Mean	Tukey HSD groups	Mean	Tukey HSD groups
MTurk	2.28 (0.28)	A	20.80 (5.37)	
Prolific	2.79 (0.29)		2.08 (3.53)	
Pollfish	2.52 (0.29)		4.94 (5.97)	A
Qualtrics	2.36 (0.29)	A	4.17 (4.97)	A

*Note:* Attention checks refer to the two bogus items and the instructional manipulation check and can take values from 0 to 3 (higher is better). The infrequency scale (IFS) score can range from 0 to 44 (lower is better). Standard errors in parentheses

Again, we also examined differences in pass rates across the three different attention checks used. As in study 1, pass rates were generally much higher across the board for the two bogus items (included in random order in the first block) than the instructional manipulation check (in the final block), which was best to differentiate between samples (Table 25 in the appendix).

Second, we checked for inattentive responses using Maniaci and Rogge’s (2014) infrequency scale (IFS). We report in Table 25 in the appendix the percentage of respondents from each platform that passed the IFS, which highlights staggering results: only 5.1% of MTurk responses met the threshold in contrast with 97.3% of Prolific responses.

Analysis showed significant differences across platforms (*F*(3, 1631) = 1229.975, *p* < .001, *f* = 1.504), with follow-up pairwise comparisons (Tukey HSD, all reported differences *p* < .05) indicating that MTurk participants scored highest on the infrequency scale, i.e., they showed the highest levels of inattentiveness (*M* = 20.8, *SD* = 5.4). Pollfish (*M* = 4.9, *SD* = 6.0) and Qualtrics respondents (*M* = 4.2, *SD* = 5.0) scored similarly to each other, while Prolific respondents scored the lowest (*M* = 2.1, *SD* = 3.5), making them the most attentive. Therefore, MTurk participants were, on average, above the cutoff point for inattentiveness (>11.5 on a scale from 0 to 44), with 94.9% of them above the cutoff point.

Third, we used a simple self-report measure (Meade & Craig, 2012), asking participants whether we should use their data. We find significant differences in the self-report measure of attentiveness (c^2^ (3) = 52.972, *p* < .001, φ = .179), with the vast majority of Prolific respondents indicating that their data should be used (98%), while it was lower for Pollfish (90%) and MTurk (89.3%), with Qualtrics being the lowest (83.7%).

Further analysis showed little correlation between the three measures (Table 6), with the infrequency scale being the most conservative measure of attentiveness (i.e., flagging a substantially higher number of respondents as potentially problematic). Given the relatively low level of correlation between attention check performance and the infrequency scale, we further examine whether this mismatch depends on the platform.

**Table 6:** Correlation between different measures of attention

	Attention checks	Infrequency scale
Attention checks	1	
Infrequency scale	– .269 (*p* < .001)	1
Self-report measure	.096 (*p* < .001)	– .043 (*p* = .083)

As Table 7 indicates, most MTurk respondents were deemed as inattentive based on the infrequency scale, although they passed at least two out of three attention checks. This performance is markedly different from the remaining platforms, with Prolific respondents demonstrating consistent results on attention checks and the infrequency measure. This presents the possibility that MTurk respondents can identify attention checks but could not identify the infrequency measure that appeared to them as another scale, which aligns with the notion that their widespread use has made typical attention checks easy to spot (Thomas & Clifford, 2017).

**Table 7:** Respondents failing the infrequency test vs. attention checks passed

Platform	Failed IFS (% of total)	Passed all 3 attention checks but failed IFS	Passed any 2 attention checks but failed IFS
MTurk	94.9%	42.9%	76.7%
Prolific	2.8%	1.5%	2.0%
Pollfish	14.0%	6.3%	12.8%
Qualtrics	9.1%	1.5%	8.4%

##### Reliability

We then compared Cronbach’s alphas to measure internal reliability for the utilized Self-Esteem and Need for Cognition Scales. Again, we find significant differences in construct reliabilities across platforms (see Table 8). More specifically, we find that Prolific produces the most reliable responses, followed by Qualtrics and Pollfish, which are equally reliable in the case of NFC, but Qualtrics delivers more reliable results for SE. Overall, our predominantly mobile-based platforms (Pollfish and Qualtrics) produced reliabilities that are above the accepted threshold (α > 0.7).

**Table 8:** Cronbach’s alphas and reliability differences between platforms

Platform	Overall α	Prolific	Pollfish	Qualtrics
	Self-Esteem			
MTurk	0.631	250.602 (< .001)	118.670 (< .001)	180.919 (< .001)
Prolific	0.935		29.779 (< .001)	7.613 (.006)
Pollfish	0.884			7.562 (.006)
Qualtrics	0.913			
	Need for Cognition			
MTurk	0.072	331.59 (< .001)	131.51 (< .001)	160.127 (< .001)
Prolific	0.891		60.226 (< .001)	43.274 (< .001)
Pollfish	0.740			1.614 (.204)
Qualtrics	0.774			

*Note:* Chi-squared tests with *p* values in parenthesis

In contrast with study 1, where we used three attention checks to determine attention, in study 2 we utilized the infrequency scale as an additional indicator of attention (inattentive respondents scored above the 11.5 threshold of the scale). As Table 9 indicates, attentive respondents (based on the infrequency scale) are significantly more reliable than inattentive respondents across platforms, demonstrating how consequential the IFS score is with all Cronbach’s alphas being significantly smaller in the inattentive subsamples and only Prolific, Pollfish and Qualtrics in the Self-Esteem measure passing the 0.7 threshold.

**Table 9:** Cronbach’s alpha values per platform, measure, and attentiveness

Platform	Overall α	Attentive	Inattentive	Chi2 (1df)	*p* value
	Self-Esteem				
MTurk	0.631	0.941 (N:22)	0.547 (N:407)	65.174	< .001
Prolific	0.935	0.937 (N:389)	0.801 (N:11)	3.909	.048
Pollfish	0.884	0.903 (N:344)	0.709 (N:56)	19.401	< .001
Qualtrics	0.913	0.920 (N:369)	0.773 (N:37)	11.830	< .001
	Need for Cognition				
MTurk	0.072	0.838 (N:22)	– 0.057 (N:407)	49.272	<.001
Prolific	0.891	0.896 (N:389)	– 0.417 (N:11)	12.069	<.001
Pollfish	0.740	0.791 (N:344)	0.291 (N:56)	21.425	< .001
Qualtrics	0.774	0.793 (N:369)	0.534 (N:37)	7.077	.008

*Note:* Attentiveness measured based on the IFS. Negative Cronbach's alpha values for NFC in the inattentive MTurk and Prolific samples suggest highly problematic response quality

##### Reproducibility

The basic pattern of all effects (conjunction fallacy, cognitive reflection test) replicated as in study 1, but the strength of the effects varied. In line with previous studies, respondents across all platforms fell prey to the conjunction fallacy. While there are significant differences in terms of the Linda problem (c^2^ (3) = 105.671, *p* < .001, φ = .254), the overall pattern was similar (ranging from 88% of Pollfish respondents to 59% of MTurk respondents regarding the less likely option to be more probable). Pairwise comparisons showed that Pollfish and MTurk were substantially different from all other platforms, while Prolific and Qualtrics were similar to each other.

For the cognitive reflection test, the differences are most dramatic (*F*(3, 1631) = 84.468, *p* < .001, *f* = .395). Participants recruited through Pollfish demonstrated a more active system 1, followed by Qualtrics participants. However, MTurk and Prolific participants demonstrated a fairly strong system 2 instead (Table 10). Pairwise comparisons indicated that these differences were all highly significant (all *p* ≤ .001).

**Table 10:** Cognitive reflection test (CRT) scores across devices used

Platform	Mean (SD)	Mean (SD): Mobile	Mean (SD): Computer
MTurk	1.83 (1.07)	0.70 (0.70)	1.90 (1.05)
Prolific	2.20 (0.98)	2.08 (1.02)	2.26 (0.96)
Pollfish	1.11 (1.06)	1.05 (1.07)	1.29 (1.02)
Qualtrics	1.39 (1.08)	1.35 (1.06)	1.51 (1.27)

*Note:* Higher mean scores indicate stronger analytical system 2 thinking. Scores range from 0 to 3

##### Social desirability

There were significant differences in terms of how participants responded to the social desirability scale across platforms (*F*(3, 1631) = 45.678, *p* < .001, *f* = .290). Follow-up analyses (Tukey HSD) showed distinct differences: Prolific participants (*M* = 2.28, *SD* = 1.67) responded in the least socially desirable way. Qualtrics participants (*M* = 3.27, *SD* = 1.83) responded in levels of social desirability comparable with MTurk participants (*M* = 3.15, *SD* = 1.09) and Pollfish participants (*M* = 3.56, *SD* = 1.82) responded in the most socially desirable way.

#### Differences across devices

##### Attention

A follow-up analysis indicated no difference in attention checks passed based on device across platforms (*F* (2, 1203) = 0.92, *p* = .399, *f* = .039). Table 11 shows the average number of passed attention checks across platforms and devices (two bogus items and one instructional manipulation check).

**Table 11:** Passed attention checks and IFS across platforms and devices

Platform	Mean (SD)	Mean (SD): Mobile	Mean (SD): Computer
MTurk	2.28 (0.79) 20.80 (5.37)	-	-
Prolific	2.79 (0.43) 2.08 (3.53)	2.73 (0.46) 1.80 (3.04)	2.82 (0.41) 2.21 (3.74)
Pollfish	2.52 (0.54) 4.94 (5.97)	2.47 (0.56) 5.43 (6.39)	2.66 (0.48) 3.37 (3.94)
Qualtrics	2.36 (0.50) 4.17 (4.97)	2.33 (0.48) 4.19 (4.96)	2.44 (0.55) 4.13 (5.02)

*Note:* The top line in each cell indicates the mean value of the three attention checks passed, and the bottom line indicates the mean infrequency score (IFS)

A closer review of the infrequency scale on a per-platform basis reveals that the device used makes no difference when Prolific and Qualtrics users are examined, while Pollfish mobile users appear less attentive based on IFS than their computer-based counterparts (*F* (1, 398) = 11.39, *p* = .001, *f* = .169).

A follow-up analysis on the self-report attention measure revealed that there was no significant platform * device interaction when it came to the self-report attention measure (c^2^ (3) = 2.816, *p* = .421, φ = .048).

##### Reliability

Follow-up analyses revealed that except for one (Prolific) out of six cases examined (Table 12), mobile users in this study provided responses that predominantly did not differ significantly in reliability from their computer-based counterparts.

**Table 12:** Cronbach’s alphas for the Self-Esteem and Need for Cognition Scales across platforms and devices

	Mobile	Computer	Chi2(1df)	*p *value
	Self-Esteem			
Prolific	0.919 (N:131)	0.942 (N:269)	4.246	.039
Pollfish	0.880 (N:305)	0.899 (N:95)	1.037	.309
Qualtrics	0.907 (N:297)	0.927 (N:109)	2.152	.142
	Need for Cognition			
Prolific	0.864 (N:131)	0.901 (N:269)	3.501	.061
Pollfish	0.724 (N:305)	0.802 (N:95)	3.417	.065
Qualtrics	0.7631 (N:297)	0.803 (N:109)	1.142	.285

##### Reproducibility

In terms of reproducibility, our analysis indicated that a respondent’s likelihood to fall prey to the conjunction fallacy across platforms did not differ based on what device they used to take part (c^2^ (2) = 2.345, *p* = .310, φ = .044).

A follow-up analysis on the cognitive reflection test revealed a significant device * platform interaction (*F* (3, 1627) = 6.37, *p* = .001, *f* = .012), with MTurk mobile respondents showing a stronger system 1 than their computer-based counterparts (*p* < .05). This means that MTurk respondents were less analytical when using mobile devices than their counterparts using computers (see Table 10).

##### Social desirability

A follow-up analysis indicated that social desirability across platforms did not differ based on the device used to participate in the study (*F* (3, 1631) = 0.571, *p* = .634, *f* = .032).

##### Context and motivation

We examined the context in which respondents participated in study 2 and their motivation for doing so. We found significant differences in participants’ level of concentration (*F*(3, 1631) = 27.170, *p* < .001, *f* = .224), level of distractions (*F*(3, 1631) = 54.828, *p* < .001, *f* = .318) and perceived time pressure (*F*(3, 1631) = 13.451, *p* < .001, *f* = .157) across platforms. Pairwise comparisons (Tukey HSD) indicated significant differences across all three measures (*p* < .05). Concentration was highest for Prolific respondents, followed by Pollfish (second highest), with Qualtrics and MTurk reporting the lowest concentration levels (final group). Prolific leads again demonstrating the lowest level of distractions and time pressure, followed by Pollfish and Qualtrics (second group), with MTurk in last place. Follow-up analyses indicated no difference between devices across platforms for the level of concentration (*F* (2, 1203) = 2.749, *p* = .064, *f* = .068), level of distractions (*F* (2, 1203) = 2.003, *p* = .135, *f* = .058), or time pressure (*F* (2, 1203) = 1.846, *p* = .158, *f* = .055).

Further, we examined participants’ motivation for taking part in the study and found significant differences across the platforms (c^2^ (12) = 442.541, *p* < .001, φ = .520). An overwhelming majority of Prolific respondents (96%) claimed to receive cash or another monetary reward, the most frequent incentive for all other platforms but at much lower levels (Table 13). All platforms differ in terms of respondent incentives (Table 26 in the appendix).

**Table 13:** Incentives per platform

Platform	Voluntary	Cash/monetary	Points/Credits	In-app
MTurk	18.5%	53.5%	16.6%	11.5%
Prolific	1.0%	96.3%	1.0%	1.8%
Pollfish	5.3%	52.8%	31.5%	10.5%
Qualtrics	5.4%	42.9%	35.7%	16.0%

We find significant differences across platforms when examining whether participants took part in the study because they had signed up to carry out such tasks (c^2^ (3) = 34.989, *p* < .001, φ = .146). Two groups emerged: 97% of Prolific and 96% of MTurk respondents claimed to have signed up, versus 90% of Pollfish and 89% of Qualtrics respondents. There were significant differences across platforms in terms of what respondents had done before completing the study (c^2^ (30) = 239.760, *p* < .001, φ = .383). Table 14 shows the distribution of activities across platforms. Admittedly, these are self-reported measures of the contextual conditions facing participants while responding to the survey, with all the limitations that this implies.

**Table 14:** Activities prior to the survey

Activity	MTurk	Prolific	Pollfish	Qualtrics
Internet browsing	21.0%	18.8%	17.8%	17.7%
Checking messages or e-mails	6.8%	12.5%	19.8%	13.6%
Engaging in social media	14.7%	7.5%	8.5%	7.9%
Watching videos or streaming content	10.0%	9.0%	8.3%	10.8%
Listening to music or podcasts	5.8%	3.0%	3.3%	4.9%
Working or studying	15.6%	6.0%	3.0%	2.5%
Playing a game	6.3%	2.8%	9.3%	20.0%
Reading news or articles	7.5%	4.3%	4.0%	3.7%
Shopping online	1.6%	2.3%	1.8%	1.5%
Completing another study or similar task	5.8%	21.8%	15.5%	11.3%
Other	4.9%	12.3%	9.0%	6.2%

##### Identifying factors of attentiveness

In line with Peer et al. (2021), we created a composite quality score focusing on attentiveness. This attentiveness composite score (ACS) includes the 11 infrequency scale (IFS) items, three attention check items (two bogus and one instructional manipulation check), and the simple self-report measure (Table 15). More specifically, the IFS items, measured on a seven-point Likert scale, were handled as attention checks with two acceptable responses (strongly agree/agree or strongly disagree/disagree depending on the question), as suggested by Meade and Craig (2012). The ACS score provided participants with a value ranging from 0 to 15, indicating the number of correctly answered items from those mentioned above. Rather than assessing a single construct, ACS is used here as a multifactorial measure reflecting the general level of attentiveness (Peer et al., 2021).

**Table 15:** ACS across platforms and devices

Platform	Mean (SD)	Mean (SD): Mobile	Mean (SD): Computer
MTurk	7.44 (2.48)	6.52 (1.97)	7.49 (2.50)
Prolific	14.28 (1.36)	14.27 (1.30)	14.29 (1.39)
Pollfish	13.01 (2.10)	12.76 (2.23)	13.79 (1.36)
Qualtrics	13.00 (1.87)	12.90 (1.89)	13.29 (1.81)

*Note:* ACS can range from 0 to 15 (higher values are better)

To unearth the factors that lead to attentiveness, we ran a regression with ACS as the dependent variable (Table 16, Model 1). We utilized the following as independent variables: platform, device used, age, gender, incentive for participating, activity before participation, ease of concentration, level of distraction, and time pressure. Moreover, we used several measures to better understand whether participants were professional survey takers and may have completed similar tasks before. Specifically, we measured usage (time spent completing such tasks or studies), study familiarity (whether they have completed a similar study in the recent past), whether they have signed up for carrying out such tasks, and the number of platforms they have signed up for. Variance inflation factors (VIFs) were calculated to assess multicollinearity among the predictor variables. The mean VIF was 1.69, and the maximum VIF was 3.91, indicating that multicollinearity is not a concern as all VIF values are below the commonly accepted threshold of 5 (James et al., 2021). This approach allows us to identify significant differences across most variables while controlling for the rest.

**Table 16:** Regression effects (eta-squared) for Attentiveness Composite Score (ACS)

Source	*df*	DV: ACS Model 1	DV: CRT Model 2	DV: ACS Model 3
Model		.714***	.189***	.726***
Platform	3	.431***	.057*	.437***
Device	1	.001	.014*	<.001
Incentive	3	.026*	.005	.023*
Activity	10	.014*	.022*	.011*
Time pressure	1	<.001	<.001	<.001
Distractions	1	.017*	<.001	.016*
Concentration	1	.001	<.001	.002
Age	1	.024*	.007	.030*
Gender	1	.010*	.003	.012*
Usage	1	.009	.006	.007
Study familiarity	1	.050*	.010*	.043*
Signed up	1	<.001	<.001	<.001
Platforms num. signed up	1	.006	<.001	.006
Cognitive Reflection Test (CRT)	1			.041*

*Note:* DV: dependent variable

***η^2^ = 0.14 (large effect), **η^2^ = 0.06 (medium effect), *η^2^ = 0.01 (small effect)

Our analysis did not find any significant difference between the device used and attentiveness, indicating that attentiveness between mobile and computer-based respondents does not differ (Table 16, Model 1). However, the platform has a large effect on attentiveness as measured by our composite score, followed by smaller effects from the incentive provided to complete the survey, age, study familiarity, distractions in the respondent’s environment, activity before engaging with the survey, and gender. Attentiveness increased with age and was generally higher among women, while distractions reduced attentiveness as expected. We performed additional pairwise comparisons using the Tukey HSD post hoc test (Table 27 in the appendix) and found that platforms differ significantly in attentiveness, except for Pollfish and Qualtrics, which belong to the same group. MTurk produces the smallest margin, indicating a significant negative effect on attentiveness, followed by Pollfish and Qualtrics. Ultimately, respondents from Prolific demonstrate the highest levels of attentiveness.

We find that incentives influence attentiveness: cash/monetary incentives lead to more attentive responses, and in-app rewards lead to the least attentive responses (Table 27 in the appendix). While voluntary participation is between these two, cash/monetary and points/credits are no different than each other, and points/credits do not differ from voluntary participation. This indicates that respondents may prefer to return to their apps rather than complete a survey. In terms of activities before engaging with our study, playing a game was the activity that led to significantly more attentive responses, while listening to music or podcasts to the least attentive (Table 27 in the appendix). However, the result of each of these two activities is similar to every other activity examined (checking messages or e-mails, social media, watching videos, working/studying, shopping online, or completing another study).

Among the measures we used to examine whether participants were professional survey takers, our regression results suggest that only study familiarity had a significant effect on ACS. Namely, not having participated in similar surveys is related to higher levels of attention (see Table 27 in the appendix). When comparing the four platforms, we find significant differences (c^2^ (6) = 195.791, *p* < .001, φ = .346), with MTurk showing by far the largest proportion of respondents having participated recently in similar studies (47.8%), while the percentages for Prolific (12%), Pollfish (14.5%) and Qualtrics (14.5%) are much lower and similar to one another. A follow-up analysis indicated that there were no differences in study familiarity across platforms based on which device was used (c^2^ (2) = 1.279, *p* = .528, φ = .033).

##### The effect of cognitive reflection

Our earlier study 2 findings corroborate study 1 findings that system 1 thinking is associated with different platforms and mobile use. However, we also explore if other factors we have examined increase the likelihood of participants engaging in system 1 thinking, which is considered "intuitive" and "fast" but might be more prone to cognitive biases and errors as it does not involve the careful analysis or reflection of system 2 thinking (Kahneman, 2011). Therefore, system 1 thinking raises concerns about less attentive responses as it may involve lower levels of working memory and attention resources (Evans & Stanovich, 2013). With mobile users tending to show stronger system 1 thinking than their computer counterparts, as demonstrated by our study 1 and other studies (King et al., 2015), a concern is raised that mobile phone use might lead to more inattentive responses. This implies that the cognitive reflection test, which measures system 1 and system 2 thinking, may mediate the relationship between mobile device use and attentiveness (Table 16, Model 2).

In order to test for the mediating role of cognitive reflection in the relationship between device use and attentiveness, we followed the joint significance approach, as it has been shown to have similar power relative to other approaches, but without the cost of inflated type I error rates (Yzerbyt et al., 2018). We hence examined the effect of all predictors on cognitive reflection (Table 16, Model 2) and, in turn, the effect of cognitive reflection along with all other predictors on attentiveness (Table 16, Model 3). On the one hand, our results indicate that CRT does indeed have a small direct effect (*η*^*2*^ = 0.042) where higher levels of system 1 thinking (lower CRT score) reduce attentiveness. On the other hand, platform, activity, device, and study familiarity have an effect on CRT. Interestingly, a pairwise comparison indicated that while platforms demonstrate the strongest overall effect on CRT, they differ significantly from each other in terms of cognitive reflection. Specifically, Pollfish respondents exhibited the highest level of system 1 thinking, followed by Qualtrics, MTurk, and then Prolific respondents, which exhibited the highest level of system 2 thinking. Additional pairwise comparisons examining the effect of activity on cognitive reflection indicated that reading news or articles and shopping online were activities associated with higher levels of system 1 thinking, while playing a game or completing another study were associated with higher levels of system 2 thinking. Finally, mobile device use and study familiarity have a small effect on CRT and are associated with system 1 thinking. Thus, while cognitive reflection appears to partially mediate the relationship between various factors (platforms, activity, study familiarity) and attentiveness, it fully mediates the relationship between device use and attentiveness. Using the MEDIATE command of Stata v.18, we confirmed CRT's mediating effect on the relationship between device and attentiveness (indirect effect coefficient = 0.174, SE = 0.038, z = 4.53).

### Summary of study 2 findings

Study 2 replicates and extends the analyses of study 1 in several ways. Regarding the platform comparison, we find that this MTurk sample performs significantly worse than the other platforms examined in terms of producing reliable measures. It is also of great concern that MTurk users appear to do reasonably well with frequently used attention checks, but the majority fail the infrequency test utilized. On the contrary, Prolific is consistent in delivering the most attentive and reliable responses. Pollfish and Qualtrics are also able to produce reliable and attentive responses. Overall, we demonstrate that attentiveness, measured by our attentiveness composite score (ACS), varies significantly across platforms, but not across devices. Further, we identified that several contextual and user-specific variables are at play (incentives, activity engaged in prior to participation, environmental distractions, age, gender, and study familiarity). We also find that mobile use has a significant, albeit small, effect on leading to system 1 thinking, which has, in turn, a negative effect on attentiveness.

## Discussion

By collecting survey responses from MTurk, Prolific, Pollfish, and Qualtrics, this paper set out to address three research questions in line with prior research (Peer et al., 2017, 2021). More specifically, we first examine whether response quality differs between platforms that depend more on mobile respondents vs. more popular, primarily computer-based platforms. Second, we examine if response quality within the same platform depends on the device used, and third, we consider whether and how the circumstances and context of the respondent, such as the impact of incentives, prior activity, environmental distractions, extent of platform use, and familiarity with similar studies, influence response quality. Below, we revisit these three questions.

When comparing the data collection platforms between our studies, in study 2, we see performance improvements, as measured by construct reliabilities and attention checks, in Qualtrics, but a performance decline in MTurk. Prolific appears to deliver consistent performance across studies, followed by Pollfish. Based on our various attentiveness measures, study 2 shows that Prolific provides the most attentive responses relative to other platforms, followed by Pollfish and then Qualtrics and MTurk. Interestingly, while most MTurk users can pass at least two attention checks, they fail considerably on the infrequency measure used. Therefore, our findings corroborate previous concerns about the quality of data collected via MTurk (e.g., Webb & Tangney, 2022). Beyond attentiveness, we found significant differences in dropout rates (Zhou & Fishbach, 2016), speed of data collection, and ease of setting up and launching a survey.

When examining the potential differences in response quality between mobile and computer-based respondents, the results from both studies predominantly confirm that within each platform, mobile-based responses are of similar quality as computer-based ones (Antoun et al., 2017). Utilizing our attentiveness composite score, the study 2 regression model, which controlled for a wide range of factors, indicated that attentiveness is not directly affected by the device used while it is largely affected by the platform. Device choice has a comparatively small but statistically significant mediated effect on our attentiveness composite score via the cognitive response of the participant.

In particular, consistent with King et al. (2015), we find that system 1 thinking is more pronounced with mobile responses. Furthermore, system 1 thinking negatively impacts attentiveness. To the extent that system 1 thinking negatively affects attentiveness and is pronounced among mobile users, researchers should consider what mode (system 1 vs. system 2) is essential for their research objectives and choose their measurement instruments accordingly. These choices should be deliberate, as research by Hauser and Schwarz (2015), for example, has shown that instructional manipulation tests, rather than just being a measure of attention, can also have unintended consequences, e.g., that they induce more reflective system 2 thinking.

When examining the role of user context and circumstances on our attentiveness composite score, we find small effects due to such factors. More specifically, we find that the incentives offered to respondents do matter, with cash/monetary incentives providing the most attentive responses, while in-app incentives lead to less attentive responses. It may be argued that tangible and direct incentives are more effective than indirect incentives that potentially interrupt the flow of another app.

The activity the user was engaged in before responding to our study is another contextual factor we examine in study 2. There, we find that playing a game prior to completing a survey is associated with higher levels of attentiveness, while listening to music or a podcast is associated with lower levels of attentiveness. We also find that distractions in the surrounding environment and familiarity with similar studies negatively affect attentiveness. Concerning our demographic controls, older and female respondents are more attentive. Researchers should consider either pre-screening or controlling for such contextual factors in their research design.

To summarize, this paper contributes to the literature on online data quality for behavioral research in three main ways. First, we find significant differences between well-established platforms (MTurk, Prolific) and more recent entrants (Qualtrics panels and Pollfish) that rely on mobile users to a much greater extent. While we join other researchers in raising concerns about MTurk (based on our study 2), the remaining three platforms produced acceptable and comparable data quality, with Prolific allowing more control over all responses received. Nonetheless, the fact that Pollfish and Qualtrics are predominantly mobile-based platforms suggests that they might reach a broader audience, a hypothesis that requires further examination.

Second, we examine the differences between computer users and mobile users within each platform and find that both yield data of comparable quality. Based on our evidence, researchers do not need to be concerned about whether their respondents take part in their studies using a mobile device or a computer, as different device usage does not appear to affect data quality adversely.

Third, we follow up on calls to focus on the context and circumstances of the respondents (Anduiza & Galais, 2017; Mavletova & Couper, 2013), and we clarify the role of incentives, prior activity, distractions, and familiarity. Notably, we confirm the association between mobile device use and system 1 thinking and, additionally, demonstrate the relationship between system 1 thinking and overall attentiveness.

## Limitations and future research

The contextual factors we measured (e.g., distractions, ability to concentrate, time pressure) were based on self-reported data, which may not fully capture the real-time conditions participants experienced. Future research could use more objective measures to better understand how context affects response quality. Our system 1 and system 2 thinking measurement relied on the cognitive reflection test (CRT), which is based on indirect measures and was not experimentally manipulated. Future studies could adopt an experimental research design to better understand how platforms, activities, and devices influence system 1 and system 2 thinking during study completion. Another area of interest for future research is the potential confounding effect of different device interfaces and pre-existing characteristics of mobile versus computer users. Mobile devices allow for different kinds of interaction compared to computers, perhaps influencing participant responses in ways unrelated to the study content. Additionally, individuals choosing to use one or the other device may have different levels of motivation, attention, or personality traits. To address these issues, we examined the personality traits (study 1) and demonstrated the role of cognitive reflection (study 2). Nonetheless, future studies should control for or experimentally manipulate interface elements (e.g., question format, navigation complexity) to better isolate the effect of the device and the characteristics of the respondent and how these might interact with response quality.

## Electronic supplementary material

Below is the link to the electronic supplementary material.

Supplementary file1 (DOCX 43 KB)

Supplementary file2 (XLSX 514 KB)

Supplementary file3 (XLSX 431 KB)

## Data Availability

Data and materials are provided as supplementary materials.

## Appendix

**Table 17:** Study 1 demographics across platforms

		MTurk	Prolific	Pollfish	Pollfish 3P	Qualtrics	Qualtrics Raw
Age	Mean (years)	39.2	33.0	45.0	38.1	45.9	45.9
	SD (years)	12.0	12.3	14.6	13.0	18.0	18.0
Gender	Male	58.3%	47.7%	47.5%	49.4%	50.6%	50.0%
	Female	41.7%	52.3%	52.5%	50.6%	49.4%	50.0%
Race	Asian	9.4%	19.9%	3.3%	3.4%	5.7%	5.6%
	Black/African American	8.4%	6.3%	7.4%	14.6%	11.7%	11.7%
	White	74.5%	60.4%	77.7%	68.7%	74.1%	73.9%
	Hispanic/Latino	5.0%	7.6%	7.4%	9.2%	4.8%	5.4%
	Multiracial	2.0%	4.3%	2.3%	1.2%	2.5%	2.4%
	Other	0.7%	1.5%	1.8%	2.9%	1.1%	1.1%
Education	Elementary School	0.0%	0.5%	0.0%	0.5%	0.5%	0.4%
	Middle School	0.2%	0.0%	1.5%	1.2%	1.1%	1.1%
	High School	21.9%	24.4%	25.3%	36.6%	34.9%	35.8%
	Vocational/Technical College	12.3%	11.0%	11.0%	23.0%	17.5%	17.3%
	Undergraduate (University)	49.6%	40.6%	38.3%	23.0%	25.2%	24.6%
	Postgraduate (University)	16.0%	23.4%	24.0%	15.7%	20.9%	20.8%
Employment	Employed for wages	75.1%	64.8%	67.3%	65.7%	63.5%	62.1%
	Self-employed	16.3%	12.8%	11.5%	14.2%	17.6%	18.3%
	Unemployed	5.6%	17.8%	13.1%	10.1%	11.6%	11.8%
	Military	0.0%	0.3%	0.3%	0.0%	0.0%	0.3%
	Homemaker	2.9%	4.3%	7.7%	10.1%	7.3%	7.5%
Income	$10,000 to $24,999	16.5%	20.2%	17.4%	26.7%	21.7%	21.8%
	$25,000 to $49,999	29.1%	23.6%	23.1%	24.7%	25.2%	25.6%
	$50,000 to $74,999	24.8%	18.6%	19.6%	16.4%	19.8%	19.6%
	$75,000 to $99,999	15.0%	13.9%	15.2%	12.3%	10.8%	10.9%
	$100,000 to $124,999	5.8%	8.7%	10.3%	6.5%	8.3%	8.0%
	$125,000 to $149,999	3.8%	5.2%	5.7%	4.5%	5.2%	5.6%
	$150,000 or more	5.0%	9.7%	8.7%	8.8%	9.0%	8.7%

**Table 18:** Mean attention checks passed across platforms and devices (Study 1)

Platform	Mean (SD)	Mean (SD): Mobile	Mean (SD): Computer
MTurk	2.80 (0.52)	-	-
Prolific	2.71 (0.48)	2.67 (0.49)	2.72 (0.48)
Pollfish	2.93 (0.29)	2.94 (0.25)	2.89 (0.36)
Pollfish 3P	1.99 (0.91)	1.99 (0.91)	1.99 (0.91)
Qualtrics	2.13 (0.85)	2.04 (0.90)	2.31 (0.72)
Qualtrics Raw	2.10 (0.88)	2.02 (0.91)	2.25 (0.79)

*Note:* There are three attention checks, therefore checks passed range from 0 to 3

**Table 19:** Pass rates per attention check across platforms (Study 1)

Platform	Bogus item 1	Bogus item 2	IMC
Overall	86.3%	95.0%	67.6%
MTurk	91.6%	96.6%	92.1%
Prolific	98.0%	99.8%	73.1%
Pollfish	95.0%	99.3%	98.5%
Pollfish 3P	72.4%	89.3%	37.3%
Qualtrics	77.8%	92.7%	42.9%
Qualtrics Raw	76.4%	91.2%	42.2%

**Table 20:** Mean values (and standard deviations) of individual differences across platforms (Study 1)

Sample	EXT	AGR	CON	NEU	OTE	SE	SC	NFC
MTurk	3.37 (1.65)	5.32 (1.35)	5.51 (1.29)	5.1 (1.54)	5.03 (1.44)	5.21 (1.37)	4.43 (1.32)	4.65 (1.56)
Prolific	3.25 (1.5)	4.94 (1.21)	5.07 (1.31)	4.37 (1.53)	5.16 (1.11)	4.66 (1.27)	3.79 (1.08)	4.87 (1.1)
Pollfish	3.72 (1.44)	5.17 (1.2)	5.6 (1.16)	4.63 (1.45)	5.07 (1.09)	5.19 (1.17)	4.08 (1.1)	4.78 (1.06)
Pollfish 3P	3.90 (1.38)	4.94 (1.33)	5.07 (1.4)	4.52 (1.44)	4.91 (1.29)	4.77 (1.23)	4.05 (1.17)	4.68 (1.09)
Qualtrics	3.66 (1.35)	5.06 (1.21)	5.16 (1.35)	4.71 (1.42)	4.79 (1.19)	4.90 (1.20)	4.01 (1.06)	4.59 (1.01)

*Note:* EXT = extraversion, AGR = agreeableness, CON = conscientiousness, NEU = neuroticism, OTE = openness to experiences, SE = self-esteem, SC = self-control, NFC = need for cognition

**Table 21:** Cronbach’s alphas across platforms, measures, and devices (Study 1)

Platform	Mobile	Computer	Chi2 (1df)	*p* value
	Self-Esteem			
Prolific	0.922 (N:83)	0.934 (N:318)	0.877	.349
Pollfish 3P	0.871 (N:300)	0.838 (N:121)	1.883	.170
Pollfish	0.910 (N:286)	0.906 (N:114)	0.083	.774
Qualtrics	0.857 (N:291)	0.857 (N:150)	0.001	.974
Qualtrics Raw	0.849 (N:312)	0.852 (N:155)	0.010	.920
	Need for Cognition			
Prolific	0.793 (N:83)	0.884 (N:318)	8.111	.004
Pollfish 3P	0.685 (N:300)	0.597 (N:121)	2.076	.150
Pollfish	0.812 (N:286)	0.809 (N:114)	0.008	.929
Qualtrics	0.619 (N:291)	0.737 (N:150)	5.880	.015
Qualtrics Raw	0.629 (N:312)	0.713 (N:155)	2.914	.088
	Self-Control			
Prolific	0.839 (N:83)	0.834 (N:318)	0.031	.860
Pollfish 3P	0.800 (N:300)	0.752 (N:121)	1.697	.193
Pollfish	0.797 (N:286)	0.822 (N:114)	0.622	.430
Qualtrics	0.694 (N:291)	0.764 (N:150)	2.961	.085
Qualtrics Raw	0.689 (N:312)	0.761 (N:155)	3.236	.072

**Table 22:** Cronbach’s alphas across platforms, measures, and exclusion policies (Study 1)

	Overall α	Strict Exclusion	Inattentive	Chi2 (1df)	*p* value
	Self-Esteem				
MTurk	0.935	0.947 (N:346)	0.811 (N:61)	26.660	<.001
Prolific	0.932	0.939 (N:289)	0.909 (N:112)	5.318	.021
Pollfish	0.909	0.914 (N:374)	0.841 (N:26)	3.294	.070
Pollfish 3P	0.863	0.923 (N:137)	0.815 (N:284)	34.065	<.001
Qualtrics	0.864	0.916 (N:168)	0.821 (N:273)	27.967	<.001
Qualtrics Raw	0.858	0.920 (N:174)	0.805 (N:293)	40.067	<.001
	Need for Cognition				
MTurk	0.925	0.946 (N:346)	0.629 (N:61)	47.571	<.001
Prolific	0.870	0.872 (N:289)	0.868 (N:112)	0.027	.870
Pollfish 3P	0.661	0.811 (N:137)	0.558 (N:284)	25.596	<.001
Pollfish	0.811	0.821 (N:374)	0.594 (N:26)	5.069	.024
Qualtrics	0.661	0.770 (N:168)	0.599 (N:273)	13.679	<.001
Qualtrics Raw	0.658	0.792 (N:174)	0.576 (N:293)	23.805	<.001
	Self-Control				
MTurk	0.879	0.892 (N:346)	0.781 (N:61)	9.429	.002
Prolific	0.835	0.839 (N:289)	0.821 (N:112)	0.400	.527
Pollfish 3P	0.787	0.858 (N:137)	0.743 (N:284)	14.958	<.001
Pollfish	0.808	0.812 (N:374)	0.770 (N:26)	0.382	.537
Qualtrics	0.720	0.768 (N:168)	0.688 (N:273)	4.035	.045
Qualtrics Raw	0.714	0.775 (N:174)	0.672 (N:293)	6.899	.009

*Note:* Inattentive respondents are those who do not meet the strict exclusion criteria

**Table 23:** Cognitive reflection test scores across platforms and devices used (Study 1)

Platform	Mean (SD)	Mean (SD): Mobile	Mean (SD): Computer
MTurk	1.89 (1.21)	-	-
Prolific	1.46 (1.25)	1.12 (1.18)	1.54 (1.26)
Pollfish	0.63 (0.96)	0.68 (1.00)	0.51 (0.84)
Pollfish 3P	0.39 (0.77)	0.34 (0.72)	0.51 (0.89)
Qualtrics	0.42 (0.82)	0.31 (0.71)	0.63 (0.97)
Qualtrics Raw	0.40 (0.80)	0.29 (0.69)	0.61 (0.96)

*Note:* Higher mean scores indicate stronger analytical system 2 thinking

**Table 24:** Study 2 demographics across platforms

		MTurk	Prolific	Pollfish	Qualtrics
Age	Mean (years)	33.0	39.6	49.2	45.1
	SD (years)	6.4	13.7	16.1	17.2
Gender	Prefer not to say	0.2%	0.3%	0.0%	0.2%
	Male	70.6%	33.8%	49.3%	48.6%
	Female	28.7%	63.3%	50.8%	51.2%
	Non-binary	0.5%	2.8%	0.0%	0.0%
Race	Arab	0.0%	0.3%	0.3%	0.2%
	Asian	6.1%	14.5%	3.0%	3.5%
	Black/African American	1.6%	18.8%	18.3%	17.2%
	White	90.7%	55.3%	70.3%	68.8%
	Hispanic	0.5%	3.8%	2.5%	5.6%
	Latino	0.2%	2.3%	0.8%	0.9%
	Multiracial	0.5%	4.0%	1.0%	2.3%
	Other	0.0%	0.8%	3.5%	0.9%
	Prefer not to say	0.5%	0.5%	0.5%	0.5%
Education	Elementary School	0.7%	0.0%	0.0%	0.0%
	Middle School	0.5%	0.5%	1.0%	0.9%
	High School	8.4%	24.5%	37.3%	41.2%
	Vocational/Technical College	10.0%	11.3%	16.5%	18.6%
	Undergraduate (University)	47.3%	44.8%	30.0%	25.1%
	Postgraduate (University)	33.1%	19.0%	15.3%	14.2%
Employment	Employed for wages	62.7%	61.3%	40.0%	38.1%
	Self-Employed	30.5%	13.3%	9.8%	10.9%
	Unemployed and looking for work	3.7%	6.0%	10.0%	8.8%
	Unemployed but not currently looking	0.9%	1.5%	2.5%	2.6%
	Homemaker	0.9%	3.0%	4.5%	5.1%
	Student	0.5%	6.5%	2.3%	4.2%
	Military	0.2%	0.8%	0.3%	0.0%
	Retired	0.5%	4.5%	23.0%	21.4%
	Unable to work	0.0%	2.5%	7.0%	7.7%
	Other	0.0%	0.8%	0.8%	1.2%
Income	$10,000 to $24,999	7.9%	14.5%	30.8%	27.9%
	$25,000 to $49,999	12.1%	22.8%	26.5%	27.0%
	$50,000 to $74,999	29.6%	19.8%	11.5%	18.8%
	$75,000 to $99,999	33.8%	15.0%	9.0%	9.1%
	$100,000 to $124,999	7.5%	9.8%	7.0%	6.5%
	$125,000 to $149,999	6.3%	6.8%	6.3%	3.5%
	$150,000 or more	2.6%	8.8%	6.8%	4.9%
	Prefer not to say	0.2%	2.8%	2.3%	2.3%

**Table 25:** Pass rates per attention check across platforms (Study 2)

Platform	Bogus item 1	Bogus item 2	IMC	IFS check
Overall	91.2%	98.4%	58.4%	68.1%
MTurk	70.2%	96.3%	61.3%	5.1%
Prolific	99.3%	99.0%	80.5%	97.3%
Pollfish	97.8%	98.8%	55.0%	86.0%
Qualtrics	99.0%	99.8%	36.9%	90.9%

*Note:* IMC = Instructional manipulation check, IFS = Infrequency scale check

**Table 26:** Pairwise chi-squared tests of the platforms in terms of incentives (Study 2)

	Prolific	Pollfish	Qualtrics
MTurk	chi2(4) = 197.5442 *p* < .001	chi2(4) = 50.3440, *p* < .001	chi2(4) = 67.1639, *p* < .001
Prolific		chi2(4) = 202.5042, *p* < .001	chi2(4) = 271.5549, *p* < .001
Pollfish			chi2(4) = 9.5732, *p* = .048

*Note:* Bonferroni correction .05/6 = .0083

**Table 27:** Margin contribution per platform, incentive, activity, and study familiarity (Study 2)

		DV: ACS		DV: CRT	
		Model 1		Model 2	
		Margin (SE)	Tukey groups	Margin (SE)	Tukey groups
Platform	MTurk	5.71 (0.43)		1.78 (0.24)	
	Prolific	11.23 (0.46)		2.08 (0.26)	
	Pollfish	10.18 (0.46)	A	1.21 (0.26)	
	Qualtrics	10.34 (0.45)	A	1.46 (0.25)	
Device	Mobile	9.28 (0.44)	A	1.47 (0.25)	
	Computer	9.45 (0.44)	A	1.80 (0.25)	
Incentive	None (Voluntary)	9.29 (0.46)	AB	1.52 (0.26)	A
	Cash	9.79 (0.44)	C	1.73 (0.25)	A
	Points or credits	9.63 (0.45)	BC	1.74 (0.26)	A
	In-app rewards	8.76 (0.45)	A	1.54 (0.26)	A
Activity	Internet browsing	9.27 (0.44)	AB	1.68 (0.25)	ABC
	Checking messages or emails	9.38 (0.45)	AB	1.74 (0.26)	BC
	Engaging in social media	9.40 (0.45)	AB	1.68 (0.26)	ABC
	Watching videos or streaming content	9.44 (0.45)	AB	1.74 (0.26)	ABC
	Listening to music or podcasts	8.96 (0.48)	A	1.46 (0.27)	ABC
	Working or studying	9.14 (0.46)	AB	1.65 (0.26)	ABC
	Playing a game	9.86 (0.44)	B	1.93 (0.25)	C
	Reading news or articles	9.21 (0.49)	AB	1.29 (0.28)	A
	Shopping online	9.33 (0.56)	AB	1.18 (0.32)	AB
	Completing another study or similar task	9.27 (0.46)	AB	1.85 (0.26)	C
	Other	9.75 (0.48)	AB	1.77 (0.27)	ABC
Study Familiarity	Yes	8.79 (0.44)		1.51 (0.25)	A
	Not sure	9.40 (0.46)		1.61 (0.26)	AB
	No	9.91 (0.44)		1.78 (0.25)	B

## Footnotes

1 Although tablets represent less than 2.5% market share worldwide (3% in the US) vs. smartphones and personal computers (StatCounter, 2022), in this paper, we use the term mobile devices to refer to both smartphones and tablets since they use the same operating systems and application stores, offering a similar end-user experience for platform respondents.

2 We thank our anonymous reviewers for drawing our attention to such factors.

3 In September 2023, a limit of 50 questions was raised to 100 questions.

4 Personal communication with the Qualtrics project manager who handled study 2.

5 We recruited MTurk respondents through the native Amazon interface rather than CloudResearch.

6 This option was available at the time of data collection but has since been discontinued by Pollfish.

7 We report all studies, measures, manipulations, and exclusions throughout.

8 See Table 17 in the appendix.

9 This is one of the occasions, in which the Qualtrics sample was significantly better than the Qualtrics Raw sample. That means by excluding the 26 responses that Qualtrics deemed of poor quality, the average passed attention checks go up. If the Qualtrics Raw sample is used in the analysis, the average is no different than the one reported for Pollfish 3P.

10 We again recruited MTurk respondents through the native Amazon interface rather than CloudResearch.

11 See Table 24 in the appendix for a summary of demographics.

12 Fees for Prolific and MTurk were adjusted from study 1 based on estimated time to complete the survey and inflation. Pollfish and Qualtrics set fees based on the number of questions.

## Code availability

No code is provided.

## References

[CR1] Anduiza, E., & Galais, C. (2017). Answering without reading: IMCs and strong satisficing in online surveys. *International Journal of Public Opinion Research,**29*(3), 497–519. 10.1093/ijpor/edw007

[CR2] Antoun, C., Couper, M. P., & Conrad, F. G. (2017). Effects of mobile versus PC web on survey response quality: A crossover experiment in a probability web panel. *Public Opinion Quarterly,**81*(S1), 280–306. 10.1093/poq/nfw088

[CR3] Berinsky, A. J., Huber, G. A., & Lenz, G. S. (2012). Evaluating online labor markets for experimental research: Amazon.com’s Mechanical Turk. *Political Analysis,**20*(3), 351–368. 10.1093/pan/mpr057

[CR4] Berinsky, A. J., Margolis, M. F., & Sances, M. W. (2016). Can we turn shirkers into workers? *Journal of Experimental Social Psychology,**66*, 20–28. 10.1016/j.jesp.2015.09.010

[CR5] Buhrmester, M., Kwang, T., & Gosling, S. D. (2011). Amazon’s Mechanical Turk: A new source of inexpensive, yet high-quality, data? *Perspectives on Psychological Science,**6*(1), 3–5. 10.1177/1745691610393980 26162106 10.1177/1745691610393980

[CR6] Campitelli, G., & Gerrans, P. (2014). Does the cognitive reflection test measure cognitive reflection? A mathematical modeling approach. *Memory and Cognition,**42*(3), 434–447. 10.3758/s13421-013-0367-9 24132723 10.3758/s13421-013-0367-9

[CR7] Chandler, J., & Paolacci, G. (2017). Lie for a dime: When most prescreening responses are honest but most study participants are impostors. *Social Psychological and Personality Science,**8*(5), 500–508. 10.1177/1948550617698203

[CR8] Chandler, J., & Shapiro, D. (2016). Conducting clinical research using crowdsourced convenience samples. *Annual Review of Clinical Psychology,**12*(1), 53–81. 10.1146/annurev-clinpsy-021815-093623 26772208 10.1146/annurev-clinpsy-021815-093623

[CR9] Chandler, J., Paolacci, G., & Mueller, P. (2013). Risks and rewards of crowdsourcing marketplaces. In P. Michelucci (Ed.), *Handbook of human computation* (pp. 377–392). Springer. 10.1007/978-1-4614-8806-4_30

[CR10] Chandler, J., Mueller, P., & Paolacci, G. (2014). Nonnaïveté among Amazon Mechanical Turk workers: Consequences and solutions for behavioral researchers. *Behavior Research Methods,**46*(1), 112–130. 10.3758/s13428-013-0365-7 23835650 10.3758/s13428-013-0365-7

[CR11] de Holanda Coelho, Lins G., Hanel, H. P. P., & Wolf, L. J. (2020). The very efficient assessment of Need for Cognition: Developing a six-item version. *Assessment,**27*(8), 1870–1885. 10.1177/1073191118793208 30095000 10.1177/1073191118793208 PMC7545655

[CR12] Evans, J. S. B. T., & Stanovich, K. E. (2013). Dual-process theories of higher cognition: Advancing the debate. *Perspectives on Psychological Science,**8*(3), 223–241. 10.1177/1745691612460685 26172965 10.1177/1745691612460685

[CR13] Ferber, R. (1977). Research by convenience. *Journal of Consumer Research,**4*(1), 57. 10.1086/208679

[CR14] Fischer, D. G., & Fick, C. (1993). Measuring social desirability: Short forms of the Marlowe-Crowne social desirability scale. *Educational and Psychological Measurement,**53*(2), 417–424. 10.1177/0013164493053002011

[CR15] Ford, J. B. (2017). Amazon’s mechanical Turk: A comment. *Journal of Advertising,**46*(1), 156–158. 10.1080/00913367.2016.1277380

[CR16] Frederick, S. (2005). Cognitive reflection and decision making. *Journal of Economic Perspectives,**19*(4), 25–42. 10.1257/089533005775196732

[CR17] Good, P. (2000). *Permutation tests*. Springer. 10.1007/978-1-4757-3235-1

[CR18] Goodman, J. K., Cryder, C. E., & Cheema, A. (2013). Data collection in a flat world: The strengths and weaknesses of mechanical Turk samples. *Journal of Behavioral Decision Making,**26*(3), 213–224. 10.1002/bdm.1753

[CR19] Goodman, J. K., & Paolacci, G. (2017). Crowdsourcing consumer research. *Journal of Consumer Research,**44*(1), 196–210. 10.1093/jcr/ucx047

[CR20] Gosling, S. D., Rentfrow, P. J., & Swann, W. B. (2003). A very brief measure of the big-five personality domains. *Journal of Research in Personality,**37*(6), 504–528. 10.1016/S0092-6566(03)00046-1

[CR21] Hakstian, A. R., & Whalen, T. E. (1976). A k-sample significance test for independent alpha coefficients. *Psychometrika,**41*(2), 219–231. 10.1007/BF02291840

[CR22] Hauser, D. J., & Schwarz, N. (2015). It’s a trap! Instructional manipulation checks prompt systematic thinking on “tricky” tasks. *SAGE Open*, *5*(2). 10.1177/2158244015584617

[CR23] Henry, P. J. (2008). College sophomores in the laboratory redux: Influences of a narrow database on social psychology’s view of the nature of prejudice. *Psychological Inquiry,**19*(2), 49–71. 10.1080/10478400802049936

[CR24] James, G., Witten, D., Hastie, T., & Tibshirani, R. (2021). *An introduction to statistical learning with applications in R* (2nd ed.). Springer. 10.1007/978-1-0716-1418-1

[CR25] John, O. P., Naumann, L. P., & Soto, C. J. (2008). Paradigm shift to the integrative big five trait taxonomy: History, measurement, and conceptual issues. In O. P. John, R. W. Robins, & L. A. Pervin (Eds.), *Handbook of personality: Theory and research* (3rd ed., pp. 114–158). Guilford Press.

[CR26] Kahneman, D. (2011). *Thinking, fast and slow*. Farrar.

[CR27] Khang, H., Kim, J. K., & Kim, Y. (2013). Self-traits and motivations as antecedents of digital media flow and addiction: The Internet, mobile phones, and video games. *Computers in Human Behavior,**29*(6), 2416–2424. 10.1016/j.chb.2013.05.027

[CR28] King, D. D., Ryan, A. M., Kantrowitz, T., Grelle, D., & Dainis, A. (2015). Mobile Internet Testing: An analysis of equivalence, individual differences, and reactions. *International Journal of Selection and Assessment,**23*(4), 382–394. 10.1111/ijsa.12122

[CR29] Knoll, J. (2016). Advertising in social media: A review of empirical evidence. *International Journal of Advertising,**35*(2), 266–300. 10.1080/02650487.2015.1021898

[CR30] Konitzer, T., Eckman, S., & Rothschild, D. (2016). Mobile as survey mode. *Proceedings of the Survey Research Methods Section* (pp. 4129–4144). American Statistical Association.

[CR31] Liberali, J. M., Reyna, V. F., Furlan, S., Stein, L. M., & Pardo, S. T. (2012). Individual differences in numeracy and cognitive reflection, with implications for biases and fallacies in probability judgment. *Journal of Behavioral Decision Making,**25*(4), 361–381. 10.1002/bdm.752 23878413 10.1002/bdm.752 PMC3716015

[CR32] Litman, L., Robinson, J., & Abberbock, T. (2017). TurkPrime.com: A versatile crowdsourcing data acquisition platform for the behavioral sciences. *Behavior Research Methods,**49*(2), 433–442. 10.3758/s13428-016-0727-z 27071389 10.3758/s13428-016-0727-z PMC5405057

[CR33] Maloney, P. W., Grawitch, M. J., & Barber, L. K. (2012). The multi-factor structure of the brief self-control scale: Discriminant validity of restraint and impulsivity. *Journal of Research in Personality,**46*(1), 111–115. 10.1016/j.jrp.2011.10.001

[CR34] Maniaci, M. R., & Rogge, R. D. (2014). Caring about carelessness: Participant inattention and its effects on research. *Journal of Research in Personality,**48*(1), 61–83. 10.1016/j.jrp.2013.09.008

[CR35] Mavletova, A., & Couper, M. P. (2013). Sensitive topics in PC web and mobile web surveys: Is there a difference? *Survey Research Methods,**7*(3), 191–205. 10.18148/srm/2013.v7i3.5458

[CR36] Meade, A. W., & Craig, S. B. (2012). Identifying careless responses in survey data. *Psychological Methods,**17*(3), 437–455. 10.1037/a0028085 22506584 10.1037/a0028085

[CR37] Mylonopoulos, N., & Theoharakis, V. (2021). Are you keeping your facebook passions and habit under control? A dual-system perspective on facebook addiction-like symptoms. *International Journal of Electronic Commerce,**25*(2), 181–203. 10.1080/10864415.2021.1887697

[CR38] Norenzayan, A., & Heine, S. J. (2005). Psychological universals: What are they and how can we know? *Psychological Bulletin,**131*(5), 763–784. 10.1037/0033-2909.131.5.763 16187859 10.1037/0033-2909.131.5.763

[CR39] Oppenheimer, D. M., Meyvis, T., & Davidenko, N. (2009). Instructional manipulation checks: Detecting satisficing to increase statistical power. *Journal of Experimental Social Psychology,**45*(4), 867–872. 10.1016/j.jesp.2009.03.009

[CR40] Palan, S., & Schitter, C. (2018). Prolific.ac — A subject pool for online experiments. *Journal of Behavioral and Experimental Finance,**17*, 22–27. 10.1016/j.jbef.2017.12.004

[CR41] Panagiotidi, M., & Overton, P. (2022). Attention deficit hyperactivity symptoms predict problematic mobile phone use. *Current Psychology,**41*(5), 2765–2771. 10.1007/s12144-020-00785-2

[CR42] Paolacci, G., & Chandler, J. (2014). Inside the turk: Understanding mechanical turk as a participant pool. *Current Directions in Psychological Science,**23*(3), 184–188. 10.1177/0963721414531598

[CR43] Paolacci, G., Chandler, J., & Ipeirotis, P. (2010). Running experiments on amazon mechanical turk. *Judgment and Decision Making,**5*(5), 411–419. 10.2139/ssrn.1626226

[CR44] Peer, E., Vosgerau, J., & Acquisti, A. (2014). Reputation as a sufficient condition for data quality on amazon mechanical turk. *Behavior Research Methods,**46*(4), 1023–1031. 10.3758/s13428-013-0434-y 24356996 10.3758/s13428-013-0434-y

[CR45] Peer, E., Brandimarte, L., Samat, S., & Acquisti, A. (2017). Beyond the Turk: Alternative platforms for crowdsourcing behavioral research. *Journal of Experimental Social Psychology,**70*(3), 153–163. 10.1016/j.jesp.2017.01.006

[CR46] Peer, E., Rothschild, D., Gordon, A., Evernden, Z., & Damer, E. (2021). Data quality of platforms and panels for online behavioral research. *Behavior Research Methods,**54*(4), 1643–1662. 10.3758/s13428-021-01694-3 34590289 10.3758/s13428-021-01694-3 PMC8480459

[CR47] Pei, W., Mayer, A., Tu, K., & Yue, C. (2020). Attention please: Your attention check questions in survey studies can be automatically answered. *The Web Conference 2020 - Proceedings of the World Wide Web Conference, WWW 2020* (pp. 1182–1193). Association for Computing Machinery. 10.1145/3366423.3380195

[CR48] Peterson, R. A. (2001). On the use of college students in social science research: Insights from a second-order meta-analysis. *Journal of Consumer Research,**28*(3), 450–461. 10.1086/323732

[CR49] Petty, R. E., & Cacioppo, J. T. (1996). Addressing disturbing and disturbed consumer behavior: Is it necessary to change the way we conduct behavioral science? *Journal of Marketing Research,**33*(1), 1–8. 10.2307/3152008

[CR50] Pollfish. (2022a). *Ho**me pag**e*. Retrieved November 16, 2022 from https://www.pollfish.com/

[CR51] Pollfish. (2022b). *Methodology*. Retrieved November 16, 2022 from https://www.pollfish.com/methodology/

[CR52] Qualtrics. (2024). *Panels & Samples*. Retrieved October 30, 2024 from https://www.qualtrics.com/uk/research-services/online-sample/

[CR53] Rosenberg, M. (1965). *Society and the adolescent self-image*. Princeton University Press.

[CR54] Sears, D. O. (1986). College sophomores in the laboratory: Influences of a narrow database on social psychology’s view of human nature. *Journal of Personality and Social Psychology,**51*(3), 515–530. 10.1037/0022-3514.51.3.515

[CR55] Sharpe Wessling, K., Huber, J., & Netzer, O. (2017). MTurk character misrepresentation: Assessment and solutions. *Journal of Consumer Research,**44*(1), 211–230. 10.1093/jcr/ucx053

[CR56] Soror, A. A., Hammer, B. I., Steelman, Z. R., Davis, F. D., & Limayem, M. M. (2015). Good habits gone bad: Explaining negative consequences associated with the use of mobile phones from a dual-systems perspective. *Information Systems Journal,**25*(4), 403–427. 10.1111/isj.12065

[CR57] Stanovich, K. E. (1999). *Who is rational? Studies of individual differences in reasoning*. Lawrence Erlbaum Associates.

[CR58] StatCounter. (2022). *Desktop vs mobile vs tablet market share worldwide*. Retrieved November 16, 2022 fom https://gs.statcounter.com/platform-market-share/desktop-mobile-tablet/worldwide

[CR59] Statista. (2021a). *Global smartphone penetration rate as share of population*. https://www.statista.com/statistics/203734/global-smartphone-penetration-per-capita-since-2005/

[CR60] Statista. (2021b). *Share of households with a computer at home worldwide*. https://www.statista.com/statistics/748551/worldwide-households-with-computer/

[CR61] Strahan, R., & Gerbasi, K. C. (1972). Short, homogeneous versions of the marlow-crowne social desirability scale. *Journal of Clinical Psychology,**28*(2), 191–193. 10.1002/1097-4679(197204)28:2%3c191::AID-JCLP2270280220%3e3.0.CO;2-G

[CR62] Thomas, K. A., & Clifford, S. (2017). Validity and mechanical Turk: An assessment of exclusion methods and interactive experiments. *Computers in Human Behavior,**77*, 184–197. 10.1016/j.chb.2017.08.038

[CR63] Thomson, K. S., & Oppenheimer, D. M. (2016). Investigating an alternate form of the cognitive reflection test. *Judgment and Decision Making,**11*(1), 99–113. 10.1017/s1930297500007622

[CR64] Tversky, A., & Kahneman, D. (1981). The framing of decisions and the psychology of choice. *Science,**211*(4481), 453–458. 10.1126/science.7455683 7455683 10.1126/science.7455683

[CR65] Tversky, A., & Kahneman, D. (1983). Extensional versus intuitive reasoning: The conjunction fallacy in probability judgment. *Psychological Review,**90*(4), 293–315. 10.1037/0033-295X.90.4.293

[CR66] Webb, M. A., & Tangney, J. P. (2022). Too good to be true: Bots and bad data from Mechanical Turk. *Perspectives on Psychological Science*. 10.1177/17456916221120027 10.1177/17456916221120027 36343213

[CR67] Weller, J. A., Dieckmann, N. F., Tusler, M., Mertz, C. K., Burns, W. J., & Peters, E. (2013). Development and testing of an abbreviated numeracy scale: A Rasch analysis approach. *Journal of Behavioral Decision Making,**26*(2), 198–212. 10.1002/bdm.1751 32313367 10.1002/bdm.1751 PMC7161838

[CR68] Yzerbyt, V. Y., Muller, D., Batailler, C., & Judd, C. M. (2018). New recommendations for testing indirect effects in mediational models: The need to report and test component paths. *Journal of Personality and Social Psychology,**115*(6), 929–943. 10.1037/pspa0000132 30550319 10.1037/pspa0000132

[CR69] Zhou, H., & Fishbach, A. (2016). The pitfall of experimenting on the web: How unattended selective attrition leads to surprising (yet false) research conclusions. *Journal of Personality and Social Psychology,**111*(4), 493–504. 10.1037/pspa0000056 27295328 10.1037/pspa0000056

