# Supplementary material for: Evaluating mobile-based data collection for crowdsourcing behavioral research
Source: Behav Res Methods. 2025 Feb 28;57(4):106. doi: 10.3758/s13428-025-02618-1 (PMC11870873; doi:10.3758/s13428-025-02618-1)
Supplement: Supplementary file 1 — Supplementary file1 (DOCX 43 KB) [file 13428_2025_2618_MOESM1_ESM.docx]

***Supplementary Materials for***

**Evaluating Mobile-Based Data Collection
for Crowdsourcing Behavioral Research**

Reverse-coded items are highlighted with an asterisk (*) throughout.

**--- STUDY 1 ---**

**Platform**

- MTurk (1)
- Prolific (2)
- Pollfish 3^rd^ party (3)
- Pollfish (4)
- Qualtrics (5)

**Device**

- Computer (0)
- Mobile (1)

**Duration**

Participation time in seconds

**Big five personality traits (Ten-item personality inventory: TIPI); Gosling et al., 2003**

Here are a number of personality traits that may or may not apply to you. Please indicate how much you agree or disagree with each statement. You should rate the extent to which the pair of traits applies to you, even if one characteristic applies more strongly than the other.

- TIPI 1: I see myself as extraverted, enthusiastic.
- TIPI 2: I see myself as reserved, quiet.*
- TIPI 3: I see myself as critical, quarrelsome.*
- TIPI 4: I see myself as sympathetic, warm.
- TIPI 5: I see myself as dependable, self-disciplined.
- TIPI 6: I see myself as disorganized, careless.*
- TIPI 7: I see myself as calm, emotionally stable.
- TIPI 8: I see myself as anxious, easily upset.*
- TIPI 9: I see myself as open to new experiences, complex.
- TIPI 10: I see myself as conventional, uncreative.*

*Participants responded on a 7-point Likert scale from strongly disagree (1) to strongly agree (7).*

*Extraversion: TIPI 1, TIPI 2; Agreeableness: TIPI 3, TIPI 4; Conscientiousness: TIPI 5, TIPI 6; Neuroticism: TIPI 7, TIPI 8; Openness to experiences: TIPI 9, TIPI 10*

**Self-esteem (Rosenberg self-esteem scale: RSES); Rosenberg, 1965**

To what extent do you agree or disagree with the following statements?

- SE 1: On the whole, I am satisfied with myself.
- SE 2: At times I think I am no good at all.*
- SE 3: I feel that I have a number of good qualities.
- SE 4: I feel I do not have much to be proud of.*
- SE 5: I am able to do things as well as most other people.
- SE 6: I certainly feel useless at times.*
- SE 7: I feel that I'm a person of worth.
- SE 8: I wish I could have more respect for myself.*
- SE 9: All in all, I am inclined to think that I am a failure.*
- SE 10: I take a positive attitude toward myself.

*Participants responded on a 7-point Likert scale from strongly disagree (1) to strongly agree (7).*

**Self-control (Brief self-control scale: BSCS); Maloney et al., 2012**

To what extent do you agree or disagree with the following statements?

- SC 1: I am good at resisting temptation.
- SC 2: I have a hard time breaking bad habits.*
- SC 3: I do certain things that are bad for me, if they are fun.*
- SC 4: I wish I had more self-discipline.*
- SC 5: People would say that I have iron self-discipline.
- SC 6: Pleasure and fun sometimes keep me from getting work done.*
- SC 7: Sometimes I can’t stop myself from doing something, even if I know it is wrong.*
- SC 8: I often act without thinking through all the alternatives.*

*Participants responded on a 7-point Likert scale from strongly disagree (1) to strongly agree (7).*

Attention check included in the self-control question block (identical response format):

- AC 1: While watching TV, I have had a fatal heart attack.

**Need for cognition (NFC scale); Lins de Holanda Coelho et al., 2020**

To what extent do you agree or disagree with the following statements?

- NFC 1: I would prefer complex to simple problems.
- NFC 2: I like to have the responsibility of handling a situation that requires a lot of thinking.
- NFC 3: Thinking is not my idea of fun.*
- NFC 4: I would rather do something that requires little thought than something that is sure to challenge my thinking abilities.*
- NFC 5: I really enjoy a task that involves coming up with new solutions to problems.
- NFC 6: I would prefer a task that is intellectual, difficult, and important to one that is somewhat important but does not require much thought.

*Participants responded on a 7-point Likert scale from strongly disagree (1) to strongly agree (7).*

**Social desirability (Brief version of the Crowne-Marlow social desirability scale); Fischer & Fick, 1993**

Listed below are a number of statements concerning personal attitudes and traits. Please read each item and decide whether the statement is true or false as it pertains to you personally.

- SD 1: I like to gossip at times.
- SD 2: There have been occasions when I took advantage of someone.
- SD 3: I'm always willing to admit it when I make a mistake.*
- SD 4: I sometimes try to get even rather than forgive and forget.
- SD 5: At times I have really insisted on having things my own way.
- SD 6: I have never been irked when people expressed ideas very different from my own.*
- SD 7: I have never deliberately said something that hurt someone's feelings.*

*Participants responded with either true (0) or false (1).*

Attention check included in the social desirability question block (identical response format):

- AC 2: I do not understand a word of English.

**Cognitive reflection test (CRT); Frederick, 2005**

Please answer the following questions.

- CRT 1: A bat and a ball cost $1.10 in total. The bat costs $1.00 more than the ball. How much does the ball cost? (cents)
- CRT 2: If it takes 5 machines 5 minutes to make 5 widgets, how long would it take 100 machines to make 100 widgets? (minutes)
- CRT 3: In a lake, there is a patch of lily pads. Every day, the patch doubles in size. If it takes 48 days for the patch to cover the entire lake, how long would it take for the patch to cover half of the lake? (days)

Answers were then coded in separate variables (CRT1_correct, CRT2_correct, CRT3_correct) as either correct (1) or incorrect (0). A unified cognitive reflection test score was then calculated (CRT).

**Framing (Asian disease problem); Tversky & Kahneman, 1981**

*Survival frame condition (coded as 1 in Frame variable)*

Imagine that the U.S. is preparing for the outbreak of an unusual disease, which is expected to kill 600 people. Two alternative programs to combat the disease have been proposed. Assume that the exact scientific estimate of the consequences of the programs are as follows:

- If Program A is adopted, 200 people will be saved.
- If Program B is adopted, there is 1/3 probability that 600 people will be saved, and 2/3 probability that no people will be saved.

Which of the two programs would you favor?

- Program A (coded as 1 in *Asian disease* variable)
- Program B (coded as 2 in *Asian disease* variable)

*Death frame condition (coded as 0 in Frame variable)*

Imagine that the U.S. is preparing for the outbreak of an unusual disease, which is expected to kill 600 people. Two alternative programs to combat the disease have been proposed. Assume that the exact scientific estimate of the consequences of the programs are as follows:

- If Program A is adopted 400 people will die.
- If Program B is adopted there is 1/3 probability that nobody will die, and 2/3 probability that 600 people will die.

Which of the two programs would you favor?

- Program A (coded as 1 in *Asian disease* variable)
- Program B (coded as 2 in *Asian disease* variable)

**Conjunction fallacy (Linda problem); Tversky & Kahneman, 1983**

Please read the following scenario:

Linda is 31 years old, single, outspoken and very bright. She majored in philosophy. As a student, she was deeply concerned with issues of discrimination and social justice, and also participated in anti-nuclear demonstrations. Which is more probable?

- Linda is a bank teller. (coded as 1 in the CF variable)
- Linda is a bank teller and is active in the feminist movement. (coded as 0 in the CF variable)

**Age**

How old are you? (in years)

**Gender**

What is your gender?

- Male (1)
- Female (2)
- Non-binary (3)
- Prefer to self-describe (4)
- Prefer not to say (0)

**Race**

What is your race?

- Arab (1)
- Asian (2)
- Black/African American (3)
- White (4)
- Hispanic (5)
- Latino (6)
- Multiracial (7)
- Other (8)
- Prefer not to say (9)

**Education**

What is your highest level of education?

- Elementary school (0)
- Middle school (1)
- High school (2)
- Vocational/technical college (3)
- Undergraduate (University) (4)
- Postgraduate (University) (5)

**Employment**

What is your current employment status?

- Employed for wages (1)
- Self-employed (2)
- Unemployed and looking for work (3)
- Unemployed but not currently looking (4)
- Homemaker (5)
- Student (6)
- Military (7)
- Retired (8)
- Unable to Work (9)
- Other (10)

**Household income**

What is your household income?

- Under $25,000 (1)
- $25,000 to $49,999 (2)
- $50,000 to $74,999 (3)
- $75,000 to $99,999 (4)
- $100,000 to $124,999 (5)
- $125,000 to $149,999 (6)
- $150,000 or more (7)
- Prefer not to say (8)

**Usage**

How much time do you spend taking part in research and surveys for rewards?

- Less than 0.5 hours per week (1)
- More than 0.5 hours and up to 1 hour per week (2)
- More than 1 hour and up to 2 hours per week (3)
- More than 2 hours and up to 4 hours per week (4)
- More than 4 hours and up to 8 hours per week (5)
- More than 8 hours and up to 20 hours per week (6)
- More than 20 hours and up to 40 hours per week (7)
- More than 40 hours per week (8)

**Instructional manipulation check (IMC)**

What was this survey about?

Below you will find several options.

Rather than selecting the one that you think is most accurate, please make sure you select Other and type Poll in the text box.

- Demographics (2)
- Personality traits (3)
- Decision-making (4)
- Politics (5)
- Employment (6)
- Other, please specify: (1)

All attention checks (AC1, AC2, IMC) were then checked and a separate variable computed for each (AC1_check, AC2_check, IMC_check) with either a pass (1) or a fail (0). A cumulative variable of passed attention checks (Number of passed ACs) was then computed.

**Scrubbed**

For the Qualtrics sample only, this variable indicates whether the data points were retained (0) or scrubbed (1) by the platform.

---STUDY II ---

**Platform**

- MTurk (1)
- Prolific (2)
- Pollfish (3)
- Qualtrics (4)

**Device**

- Computer (0)
- Mobile (1)

**Duration**

Participation time in seconds

**Self-esteem (Rosenberg self-esteem scale: RSES); Rosenberg, 1965**

To what extent do you agree or disagree with the following statements?

- SE 1: On the whole, I am satisfied with myself.
- SE 2: At times I think I am no good at all.*
- SE 3: I feel that I have a number of good qualities.
- SE 4: I am able to do things as well as most other people.
- SE 5: I feel I do not have much to be proud of.*
- SE 6: I certainly feel useless at times.*
- SE 7: I feel that I'm a person of worth.
- SE 8: I wish I could have more respect for myself.*
- SE 9: All in all, I am inclined to think that I am a failure.*
- SE 10: I take a positive attitude toward myself.

*Participants responded on a 7-point Likert scale from strongly disagree (1) to strongly agree (7).*

Attention check included in the social desirability question block (identical response format):

- AC 1: I have 17 fingers on my left hand.

**Need for cognition (NFC scale); Lins de Holanda Coelho et al., 2020**

To what extent do you agree or disagree with the following statements?

- NFC 1: I would prefer complex to simple problems.
- NFC 2: I like to have the responsibility of handling a situation that requires a lot of thinking.
- NFC 3: Thinking is not my idea of fun.*
- NFC 4: I would rather do something that requires little thought than something that is sure to challenge my thinking abilities.*
- NFC 5: I really enjoy a task that involves coming up with new solutions to problems.
- NFC 6: I would prefer a task that is intellectual, difficult, and important to one that is somewhat important but does not require much thought.

*Participants responded on a 7-point Likert scale from strongly disagree (1) to strongly agree (7).*

**Social desirability (Brief version of the Crowne-Marlow social desirability scale); Fischer & Fick, 1993**

Listed below are a number of statements concerning personal attitudes and traits. Please read each item and decide whether the statement is true or false as it pertains to you personally.

- SD 1: I like to gossip at times.
- SD 2: There have been occasions when I took advantage of someone.
- SD 3: I'm always willing to admit it when I make a mistake.*
- SD 4: I sometimes try to get even rather than forgive and forget.
- SD 5: At times I have really insisted on having things my own way.
- SD 6: I have never been irked when people expressed ideas very different from my own.*
- SD 7: I have never deliberately said something that hurt someone's feelings.*

*Participants responded with either true (0) or false (1).*

Attention check included in the social desirability question block (identical response format):

- AC 2: I was born on planet Earth.

**Infrequency scale (IFS); Maniaci & Rogge, 2014**

To what extent is the following statement true for/to you?

- IFS1: I don't like getting speeding tickets.
- IFS2: It feels good to be appreciated.
- IFS3: I'd rather be hated than loved.*
- IFS4: I enjoy the music of Marlene Sandersfield.*
- IFS5: My favorite subject is agronomy.*
- IFS6: I don't like being ridiculed or humiliated.
- IFS7: I enjoy receiving telemarketers' calls.*
- IFS8: My main interests are coin collecting and interpretive dancing.*
- IFS9: I'd be happy if I won the lottery.
- IFS10: I love going to the DMV (Department of Motor Vehicles).*
- IFS11: I look forward to my time off.

*Participants responded on a 5-point Likert scale from very true (0) to not at all true (4).*

Each IFS statement was coded as a separate variable (IFS1_check to IFS11_check) with either a pass (1), if the response is 0 or 1, or a fail (0), if the response is 2 or higher.

Then $IFS_{passed}=\sum_{i=1}^{11} {IFS}_{i}\_check$

**Cognitive reflection test (CRT); Thomson & Oppenheimer, 2016**

Please answer the following questions.

- CRT 1: If you’re running a race and you pass the person in second place, what place are you in?
- CRT 2: A farmer had 15 sheep and all but 8 died. How many are left?
- CRT 3: Emily’s father has three daughters. The first two are named April and May. What is the third daughter’s name?

Answers were then coded in separate variables (CRT1_correct, CRT2_correct, CRT3_correct) as either correct (1) or incorrect (0). A unified cognitive reflection test score was then calculated (CRT).

**Conjunction fallacy (Linda problem); Tversky & Kahneman, 1983**

Please read the following scenario:

Linda is 31 years old, single, outspoken and very bright. She majored in philosophy. As a student, she was deeply concerned with issues of discrimination and social justice, and also participated in anti-nuclear demonstrations. Which is more probable?

- Linda is a bank teller. (coded as 1 in the CF variable)
- Linda is a bank teller and is active in the feminist movement. (coded as 0 in the CF variable)

**Concentration**

How easy is it for you to concentrate at the moment?

*Participants responded on a 5-point Likert scale from very difficult (1) to very easy (5).*

**Time pressure**

How would you describe your time constraints right now?

- Under significant time pressure (1)
- Somewhat rushed (2)
- Neither rushed nor leisurely (3)
- Relatively leisurely (4)
- At leisure, with no time constraints (5)

**Distractions**

Please rate the level of distractions in your current environment (e.g. noise, music, television, conversations).

- Extreme level of distractions (1)
- High level of distractions (2)
- Moderate distractions (3)
- Slight distractions (4)
- No distractions (5)

**Activity**

Please select the activity you were engaged in on your device just before starting this study.

- Browsing the internet (1)
- Checking messages or emails (2)
- Engaging in social media (3)
- Watching videos or streaming content (4)
- Listening to music or podcasts (5)
- Working or studying (6)
- Playing a game (7)
- Reading news or articles (8)
- Shopping online (9)
- Completing another study or similar task (10)
- Other, please specify (11)

**Incentive**

What type of incentive do you receive for participating in this study?

- None (voluntary participation) (0)
- Cash or monetary reward (1)
- Points or credits (for goods/services) (2)
- In-app rewards (extra features or bonus) (3)
- Other, please specify (4)

**Volunteer**

I am responding to this study, because I have signed up to carry out such tasks.

*Participants responded with either No (0) or Yes (1).*

**Age**

How old are you? (in years)

**Gender**

What is your gender?

- Male (1)
- Female (2)
- Non-binary (3)
- Prefer to self-describe (4)
- Prefer not to say (0)

**Race**

What is your race?

- Arab (1)
- Asian (2)
- Black/African American (3)
- White (4)
- Hispanic (5)
- Latino (6)
- Multiracial (7)
- Other (8)
- Prefer not to say (9)

**Education**

What is your highest level of education?

- Elementary school (0)
- Middle school (1)
- High school (2)
- Vocational/technical college (3)
- Undergraduate (University) (4)
- Postgraduate (University) (5)

**Employment**

What is your current employment status?

- Employed for wages (1)
- Self-employed (2)
- Unemployed and looking for work (3)
- Unemployed but not currently looking (4)
- Homemaker (5)
- Student (6)
- Military (7)
- Retired (8)
- Unable to Work (9)
- Other (10)

**Household income**

What is your household income?

- Under $25,000 (1)
- $25,000 to $49,999 (2)
- $50,000 to $74,999 (3)
- $75,000 to $99,999 (4)
- $100,000 to $124,999 (5)
- $125,000 to $149,999 (6)
- $150,000 or more (7)
- Prefer not to say (8)

**Usage**

How much time do you spend taking part in research and surveys for rewards?

- Less than 0.5 hours per week (1)
- More than 0.5 hours and up to 1 hour per week (2)
- More than 1 hour and up to 2 hours per week (3)
- More than 2 hours and up to 4 hours per week (4)
- More than 4 hours and up to 8 hours per week (5)
- More than 8 hours and up to 20 hours per week (6)
- More than 20 hours and up to 40 hours per week (7)
- More than 40 hours per week (8)

**Platforms**

How many platforms do you use to take part in studies such as this one?
*If you don't use any platforms, then please enter 0. (numerical response)*

**Study familiarity**

Have you completed a study similar to this study in the past several months?

- Yes (2)
- Not sure (1)
- No (0)

**Instructional manipulation check (IMC)**

What was this survey about?

Below you will find several options. Rather than selecting the one that you think is most accurate, please make sure you select Other and type Poll in the text box.

- Demographics (2)
- Personality traits (3)
- Decision-making (4)
- Politics (5)
- Employment (6)
- Other, please specify: (1)

All attention checks (AC1, AC2, IMC) were then checked and a separate variable computed for each (AC1_check, AC2_check, IMC_check) with either a pass (1) or a fail (0).

**Honesty**

In your honest opinion, should we use your data?

*Participants responded with either No (0) or Yes (1).*

**The Attentiveness Composite Score (ACS)** was calculated as the sum of AC1_check, AC2_check, IMC_check, IFS_passed, Honesty
